# The Association Between Life Events and Incidence of Hypertension Among Government Employees in China: A Prospective Cohort Study

**DOI:** 10.3389/fpsyg.2022.822610

**Published:** 2022-05-30

**Authors:** Feiyun Ouyang, Jun He, Xunjie Cheng, Dan Qiu, Ling Li, Joseph Benjamin Bangura, Yanyin Duan, Dan Luo, Shuiyuan Xiao

**Affiliations:** ^1^Department of Social Medicine and Health Management, Xiangya School of Public Health, Central South University, Changsha, China; ^2^Hunan Provincial Key Laboratory of Clinical Epidemiology, Changsha, China; ^3^Department of Geriatric Medicine, Xiangya Hospital, Central South University, Changsha, China; ^4^Department of Occupational and Environmental Health, Xiangya School of Public Health, Central South University, Changsha, China

**Keywords:** life events, hypertension, dose-effect relationship, government employee, prospective cohort study

## Abstract

**Background:**

Hypertension (HTN) is a global public health concern. However, the association between life events (LEs) and HTN is complex. Thus, we conducted a prospective cohort study to explore this complex association.

**Methods:**

A total of 8,077 government employees without HTN were recruited through cluster sampling between 2018 and 2019 in Hunan Province, China. At baseline, information regarding sociodemographic characteristics, LEs, and behavioral factors was collected. After the 1-year follow-up, the participants were revisited to obtain the HTN diagnosis. Crude and adjusted Poisson regression models were constructed to calculate the incidence rate ratios (IRRs) and 95% confidence intervals (CIs). Cubic regression spline models were used to visualize the trends between LEs and HTN IRRs. Interactive and subgroup analyses were also performed.

**Results:**

The 1-year HTN incidence rate among government employees in Hunan province was 4.30% (95% CI: 3.86–4.74%). LEs were associated with a higher HTN risk (IRR, 1.02; 95% CI, 1.00–1.04). When calculating positive and negative LEs scores separately, only the latter was a risk factor for HTN incidence (IRR, 1.04; 95% CI, 1.03–1.06); conversely, positive LEs reduced the risk (IRR, 0.90; 95% CI, 0.85–0.96). Compared with patients in the lowest quartile of LEs score, those in quartiles two (IRR, 1.28; 95% CI, 0.96–1.71), three (IRR, 1.43; 95% CI, 1.04–1.96), and four (IRR, 1.73; 95% CI, 1.26–2.37) were at progressively higher risk. In restricted spline curves, a non-linear association was noted between LEs and HTN risk. Regarding the subcategories of LEs, work-related LEs, personal LEs, and all subcategories of negative LEs were associated with an increased risk of HTN. However, among positive LEs, only the family-related cases were associated with a lower risk of HTN.

**Conclusion:**

LEs had a non-linear association with an increased risk of HTN. Negative LEs were risk factors for HTN incidence, whereas positive LEs reduced the risk of HTN. Thus, the importance of LEs should be highlighted in the development of HTN prevention strategies and initiatives.

## Introduction

Hypertension (HTN) is a leading risk factor for premature death and an important public health concern (GBD 2019 Risk Factors Collaborators, 2020). The global prevalence rate for HTN was 31.1% in 2010, affecting approximately 1.39 billion individuals worldwide (Mills et al., 2016). In China, nearly half (44.7%) of adults aged 35-75 years had HTN in 2017 (Lu et al., 2017). Moreover, the number is increasing consistently with the aging population and the diversification of risk factors (Mills et al., 2020). HTN is a multi-etiological chronic disease resulting from a combination of genetic, environmental, and social factors (Carey et al., 2018). Studies have shown that among all risk factors, inconsistencies are prevalent in the association between life events (LEs) and HTN (Edwards, 1995; Sparrenberger et al., 2008; Hassoun et al., 2015; Wilson-Genderson et al., 2017).

LEs have been defined as objective occurrences that require adjustment in daily life (Holmes and Rahe, 1967; Cohen et al., 2019). They are regular events that occur irregularly, such as the birth of a child or job loss. In the early twentieth century, researchers found that negative LEs were related to increased blood pressure (BP) (Myers and Miles, 1981), whereas positive LEs may act as a buffer against it (Svensson and Theorell, 1983). However, some researchers have achieved contradictory results (Svensson and Theorell, 1983; Odia and Uzogara, 1988; Edwards, 1995). These conflicting findings may be attributed to factors such as methodological weaknesses in the study design and small sample size.

Since the twentieth century, researchers have conducted a series of laboratory experiments and psychophysiological studies to analyze the effect of LEs on BP regulation and the onset of HTN. Several pathways were proposed to explain the underlying mechanisms, mainly the hypothalamic-pituitary-adrenal (HPA) axis, autonomic nervous, and immune systems. Neurohormonal models suggest that the hypothalamus synthesizes corticotropin-releasing factor (CRF) and vasopressin in response to LEs-induced stress (Osborne et al., 2020). CRF stimulates the anterior pituitary to release the adrenocorticotropic hormone, which is associated with increased BP. In addition to the HPA axis, stress-induced changes in the sympathetic (SNS) and parasympathetic nervous systems (PSNS) also play pivotal roles in the onset of HTN (Johansson et al., 1999). The dysregulation of the immune system was identified as another key physiological consequence of LEs (Lee et al., 2004). LEs-induced stress can increase the release of specific inflammatory cytokines, contributing to the dysfunction of the vascular endothelium and impairments in BP regulation. Conversely, positive LEs are related to decreased neuroendocrine and inflammatory levels through positive emotions (Steptoe et al., 2005). However, laboratory experiments and psychophysiological studies do not simulate the natural process of the formation of HTN. Thus, more epidemiological evidence is needed to validate the relationship between LEs and HTN.

In the twenty-first century, several observational studies have been performed to explore the relationship between LEs and HTN in real-world settings (Sparrenberger et al., 2008; Chai et al., 2015; Karatzias et al., 2015; Sammul et al., 2019). However, these studies are limited as the strength of the evidence varied widely and the conclusions remained unclear. Notably, most previous studies used a cross-sectional design, thus, it was difficult to infer the cause-and-effect relationship between LEs and the onset of HTN (Sparrenberger et al., 2008; Chai et al., 2015; Karatzias et al., 2015). Moreover, cumulative exposure to LEs is associated with an increased risk of HTN, thus suggesting a dose-effect relationship (Cohen et al., 2019). However, it is unclear whether the relationship between LEs and HTN risk is linear or non-linear.

Another limitation is that most previous studies have not examined the strength of the association between LEs and HTN across different contexts (e.g., at home vs. at work). Specific comparisons between these factors would be more robust if several contexts were included in the same study (Sammul et al., 2019). Furthermore, the impact of an LE depends on its positive or negative nature. Previous research focused mainly on positive and negative LEs in isolation (Karatzias et al., 2015). However, in reality, they often exist concurrently and have different effects on BP. Thus, further research is needed to comprehensively elucidate the relationship between LEs and the onset of HTN.

Chinese people are faced with various pressures from family and work owing to the developing economy and society. In particular, government employees constitute a significant proportion of the workforce. Generally, government employees are physically inactive, and therefore, are more susceptible to developing chronic diseases than the general population (Lallukka et al., 2008; Sun et al., 2016; He et al., 2020). However, evidence regarding the association between LEs and HTN among this particular population is lacking. Thus, using data from a prospective cohort of government employees, this study aimed to investigate the association and dose-effect relationship between LEs and the incidence of HTN among this population.

## Participants and Methods

### Study Design and Participants

This study was based on data from a cohort study on chronic diseases among government employees from five cities in Hunan Province, China. Detailed information on the study design and data collection process has been published previously (Li et al., 2021; Qiu et al., 2021). The participants were recruited consecutively through multistage, stratified cluster sampling between January 2018 and November 2019, and were then revisited during the 1-year follow-up. First, five cities (i.e., Changsha, Zhuzhou, Huaihua, Xiangtan, and Changde) in Hunan Province were selected based on their levels of economic development and geographic location. Second, several public departments or state-owned enterprises in each city were selected to participate in the cohort based on the departmental organizational skills, population stability, and scale. Third, all employees in the selected departments were invited to a general hospital in the city to complete the necessary research process. Informed consent was obtained from each participant prior to data collection. This study was approved by the Institutional Review Board of Xiangya School of Public Health, Central South University (No. XYGW–2016–10).

**FIGURE 1:**
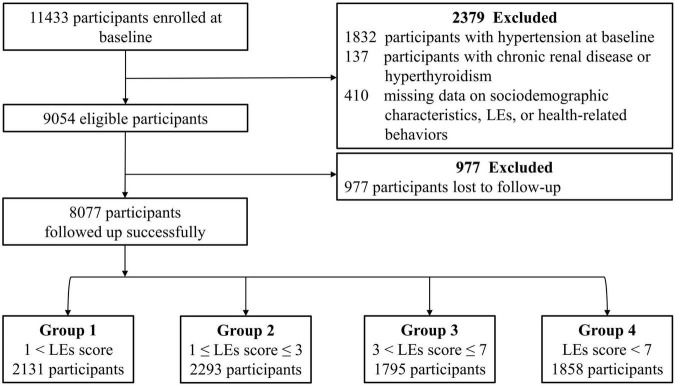
Flowchart illustrating the inclusion of participants. LEs, life events.

A total of 11,433 participants were enrolled in this cohort. At baseline, 1,832 participants were diagnosed with HTN, and 137 were diagnosed with chronic renal disease or hyperthyroidism. In addition, 410 did not provide complete information regarding their demographic characteristics, health-related behaviors, or experience of LEs. As a result, 9,054 participants were eligible for this study. However, 977 participants were lost during the 1-year follow-up; therefore, 8,077 participants were included in the final analysis. This process is illustrated in detail in the flow diagram shown in Figure 1.

### Assessment of Life Events

In this study, the baseline LEs score was evaluated using a scale designed by Desen Yang based on the Chinese cultural context (Yang and Zhang, 1993). The scale includes 48 common LEs categorized as work-related, family-related, or other personal events (see Supplementary Table 1). Since its development, this LEs scale has been used widely across research (Zhu et al., 1998; Liu et al., 2009; Chen et al., 2012; Xu et al., 2013; Wang et al., 2017). In this study, participants were asked whether they had experienced one or more of the listed LE(s) during the 12 months before the baseline period. If so, further inquiry was made regarding each event’s severity score, which was divided into four levels, namely, 1 = mild impact, 2 = moderate impact, 3 = severe impact, and 4 = extremely severe impact. The total LEs score was calculated by summing the severity scores of all events that the participant had experienced.

Based on previous studies (Liu et al., 2009; Chen et al., 2012; Li et al., 2021), LEs were classified as “positive” or “negative” on this scale (see Supplementary Table 2). The scores for positive and negative LEs were calculated based on the summation of the severity scores of the respective positive and negative LEs.

To study the relationship between different types of events and HTN, LEs were further classified into three categories: work-related, family-related, and other personal LEs (see Supplementary Table 2). For each category, the total, positive, and negative LEs scores were calculated accordingly.

### Measurement of Blood Pressure

BP was measured according to the procedures for clinical BP measurement and was performed by qualified nurses or physicians from the designated hospitals in this cohort study (Whelton et al., 2018).

Before the measurement, participants were required to rest for at least 5 min in a quiet environment. During the measurement, participants were asked to maintain a seated position with one arm exposed, placing their elbow on the same level as the heart. Systolic BP (SBP) and diastolic BP (DBP) were measured twice at 1–2-min intervals using an electronic manometer. The average value of the two readings was considered the final BP record. When the first two readings differed by >5 mmHg, a third measurement was conducted, and their mean value was considered the final BP record.

### Diagnosis of Hypertension

Participants with primary HTN were diagnosed by qualified physicians in the designated hospitals of this cohort study. HTN is defined according to the *2018 Chinese Guidelines for Prevention and Treatment of Hypertension* as follows: (1) Self-reported HTN with diagnostic records, (2) current antihypertensive treatment, and (3) clinic SBP ≥ 140 mmHg and/or DBP ≥ 90 mmHg without the use of antihypertensive medications at three visits on different days (Joint Committee for Guideline Revision, 2019).

Physicians identified masked HTN and white-coat HTN based on out-of-office BP measurements. Secondary forms of HTN were also differentiated according to the diagnosis of other diseases, laboratory tests results, and medical images.

### Assessment of Covariates

Sociodemographic characteristics (age, sex, marital status, educational level, occupation, and position title), health-related behaviors (smoking, alcohol consumption, and physical activity), and family history of patients diagnosed with HTN were investigated using standardized e-questionnaires.

The respondents were provided with three options for each characteristic, namely, marital status (unmarried, married, and divorced/widowed), educational level (high school or below, bachelor’s degree, and postgraduate or above), and position title (junior professional staff or below, intermediate professional staff, and senior professional staff or higher). Respondents’ occupations were classified into five categories: government officials, teachers or researchers, police, medical staff, and other government employees. Regarding health-related behaviors, respondents were asked about their frequency of participation in physical activities (less than once per week, 1-2 times per week, or more than 3 times per week), smoking status (non-smoker, current smoker, or former smoker), and alcohol consumption status (non-drinker, current drinker, or former drinker).

Height and weight were measured using calibrated gauges. The body mass index (BMI, kg/m^2^) was calculated by dividing weight (kg) by squared height (m). Chronic renal disease, hyperthyroidism, diabetes, hyperuricemia, and liver cirrhosis were determined based on diagnoses from physicians.

### Data Quality Management

A trained quality control team monitored the data-collection process and issued daily quality control reports, adhering to a quality control handbook. Furthermore, the research supervisor conducted regular quality assessments.

### Statistical Analysis

The participants’ general characteristics were described using mean ± standard deviation or median (interquartile range) for continuous variables and number (percentage) for categorical variables. In this study, *t*-tests were performed for normally distributed continuous variables, Wilcoxon rank-sum tests for skewed distributed continuous and ordinal variables, and chi-square tests for categorical variables to compare the differences in general characteristics between included and excluded individuals.

The included participants were divided into four groups according to the quartiles of their LEs scores. The participants’ general characteristics were compared across groups using analysis of variance for continuous variables, chi-square tests for categorical variables, and Kruskal-Wallis *H*-tests for ordinal categorical variables. The HTN incidence rates (IRs) and 95% confidence intervals (CIs) were estimated based on the sample means and standard errors.

The crude, age- and sex-adjusted, and multivariable Poisson models were constructed to calculate the incidence rate ratios (IRRs) and 95% CIs to explore the association between continuous LE scores and HTN. In the multivariable Poisson models, age, sex, BMI, baseline SBP and DBP, psychosocial factors (marital status, educational level, occupation, and position title), health-related behaviors (smoking, alcohol consumption, and physical activity), and family history of HTN were incorporated as covariables. First, HTN IRRs were calculated according to the total LEs score. Thereafter, the positive and negative LEs scores were calculated separately and added concurrently to the Poisson models to explore the difference in the risk of HTN. Regarding the subcategories of LEs, HTN IRRs of work-related, family-related, and other personal LEs were calculated in different Poisson models separately.

Regarding LE score quartiles, the HTN IRR compared each of the upper LEs score quartiles with the lowest LEs score quartile (the reference group), and calculations were made using multivariable Poisson models. Furthermore, the models with restricted cubic spline function, which had five knots located at the 5th, 27.5th, 50th, 72.5th, and 95th percentiles of the distribution of LEs score, were used to analyze the curvilinear dose-effect relationship between LEs and the incidence of HTN visually. Interactive and subgroup analyses were performed to assess the heterogeneity of the effects across the subgroups. For the sensitivity analysis, participants with diabetes, hyperuricemia, and/or liver cirrhosis were excluded from the Poisson models to verify the robustness of the results.

The analyses were performed using R version 4.1.0. All tests were two-sided. Differences were considered statistically significant at *P* < 0.05. In the subgroup analysis, the significance levels were adjusted using the Bonferroni method to minimize Type I error.

## Results

### Characteristics of Study Participants

The median age of the 8,077 participants was 35.7 (30.5, 43.4) years, 31.8% were males, 19.0% were unmarried, 34.2% had a postgraduate degree or higher, and 52.8% were intermediate (36.9%) or senior (15.9%) professional staff. Compared with the excluded individuals, the included individuals were younger; had lower BMI and baseline SBP and DBP; and tended to be female. A detailed comparison between the included and excluded participants is shown in Supplementary Table 3.

Participants were then divided into four groups according to quartiles of the LEs score, namely, group one (LEs score < 1 point), group two (LEs score between 1 and 3 points), group three (LEs score between 3 and 7 points), and group four (LEs score > 7 points). The comparisons of the demographic characteristics across the groups are shown in Table 1. The median age decreased with the increase in quartiles of LEs score (40.0, 36.0, 34.0, and 33.0 years). Differences in BMI, smoking, and alcohol consumption were not statistically significant among the groups. However, other characteristics displayed irregular differences across the groups (see Table 1).

**TABLE 1:** Participants’ characteristics according to quartiles of life events (LEs) score.

Variables	Whole cohort	Group 1	Group 2	Group 3	Group 4	*P*-value
Number of participants	8,077	2,131	2,293	1,795	1,858	
Age (years)	35.7 (30.5, 43.4)	40.0 (33.0, 48.0)	36.0 (31.0, 44.5)	34.0 (30.0, 40.0)	33.0 (29.4, 37.7)	< 0.001*
BMI (kg/m^2^)	22.5 (20.5, 24.8)	22.6 (20.7, 24.9)	22.4 (20.3, 24.8)	22.5 (20.6, 24.9)	22.4 (20.4, 24.7)	0.098
Baseline SBP (mmHg)	114.0 (107.0, 123.0)	116.0 (108.0, 125.0)	114.0 (107.0, 123.0)	114.0 (106.0, 123.0)	113.0 (105.0, 122.0)	< 0.001*
Baseline DBP (mmHg)	69.0 (63.0, 76.0)	70.0 (64.0, 77.0)	69.0 (64.0, 76.0)	69.0 (63.0, 76.0)	68.0 (62.0, 75.0)	< 0.001*
**Sex**						
Male	2,569 (31.8)	744 (34.9)	698 (30.4)	605 (33.7)	522 (28.1)	< 0.001*
Female	5,508 (68.2)	1,387 (65.1)	1,595 (69.6)	1,190 (66.3)	1,336 (71.9)	
**Marital status**						
Unmarried	1,538 (19.0)	340 (16.0)	477 (20.8)	386 (21.5)	335 (18.0)	< 0.001*
Married	6,362 (78.8)	1,743 (81.8)	1,773 (77.3)	1,372 (76.4)	1,474 (79.3)	
Divorced or widowed	177 (2.2)	48 (2.3)	43 (1.9)	37 (2.1)	49 (2.6)	
**Educational level**						
High school or below	250 (3.1)	104 (4.9)	79 (3.4)	37 (2.1)	30 (1.6)	0.003*
Bachelor	5,066 (62.7)	1,333 (62.6)	1,432 (62.5)	1,102 (61.4)	1,199 (64.5)	
Postgraduate or above	2,761 (34.2)	694 (32.6)	782 (34.1)	656 (36.5)	629 (33.9)	
**Occupation**						
Government officials	274 (3.4)	71 (3.3)	96 (4.2)	54 (3.0)	53 (2.9)	< 0.001*
Teachers or researchers	1,672 (20.7)	507 (23.8)	488 (21.3)	357 (19.9)	320 (17.2)	
Police	341 (4.2)	81 (3.8)	91 (4.0)	76 (4.2)	93 (5.0)	
Medical staff	5,035 (62.3)	1,243 (58.3)	1,413 (61.6)	1,145 (63.8)	1,234 (66.4)	
Other government employees	755 (9.3)	229 (10.7)	205 (8.9)	163 (9.1)	158 (8.5)	
**Position title**						
Junior professional staff	3,814 (47.2)	825 (38.7)	1,060 (46.2)	902 (50.3)	1,027 (55.3)	< 0.001*
Intermediate professional staff	2,982 (36.9)	823 (38.6)	867 (37.8)	632 (35.2)	660 (35.5)	
Senior professional staff	1,281 (15.9)	483 (22.7)	366 (16.0)	261 (14.5)	171 (9.2)	
**Physical activity**						
< 1/week	3,865 (47.9)	845 (39.7)	1,028 (44.8)	929 (51.8)	1,063 (57.2)	< 0.001*
1–2/week	2,125 (26.3)	558 (26.2)	646 (28.2)	474 (26.4)	447 (24.1)	
> 3/week	2,087 (25.8)	728 (34.2)	619 (27.0)	392 (21.8)	348 (18.7)	
**Smoking**						
Non-smokers	7,254 (89.8)	1,913 (89.8)	2,045 (89.2)	1,620 (90.3)	1,676 (90.2)	0.068
Current smokers	723 (9.0)	179 (8.4)	221 (9.6)	161 (9.0)	162 (8.7)	
Former smokers	100 (1.2)	39 (1.8)	27 (1.2)	14 (0.8)	20 (1.1)	
**Alcohol consumption**						
Non-drinkers	6,806 (84.3)	1,774 (83.2)	1,952 (85.1)	1,512 (84.2)	1,568 (84.4)	0.413
Current drinkers	1,219 (15.1)	337 (15.8)	330 (14.4)	273 (15.2)	279 (15.0)	
Former drinkers	52 (0.6)	20 (0.9)	11 (0.5)	10 (0.6)	11 (0.6)	
**Family history of hypertension**						
With family history of hypertension	5,408 (67.0)	1,541 (72.3)	1,500 (65.4)	1,165 (64.9)	1,202 (64.7)	< 0.001*
Without family history of hypertension	2,669 (33.0)	590 (27.7)	793 (34.6)	630 (35.1)	656 (35.3)	

*P < 0.05. The entire cohort was divided into four groups according to quartiles of the LEs score: group 1 (LEs score < 1 point), group 2 (LEs score between 1 and 3 points), group 3 (LEs score between 3 and 7 points), and group 4 (LEs score > 7 points). Age, BMI, and baseline SBP and DBP are presented as median (interquartile range). The other variables are presented as number (percentage). SBP, systolic blood pressure; DBP, diastolic blood pressure; BMI, body mass index; kg, kilogram; m, meter.

### Hypertension Incidence Rates Across Study Groups

After the 1-year follow-up, 350 participants had developed HTN (IR: 4.30%, 95% CI: 3.86–4.74%). The HTN IRs across the four groups were 4.00% (95% CI: 3.17–4.83%), 4.40% (95% CI: 3.56–5.24%), 4.30% (95%CI: 3.36–5.24%), and 4.70% (95% CI: 3.74–5.66%), respectively.

### Hypertension Incidence Rate Ratios Association With Life Events Score

In the multivariable Poisson model, HTN IRR was 1.02 (95% CI: 1.00–1.04) for the total score of all LEs. When calculating positive and negative LEs scores separately, HTN IRRs were 0.90 (95% CI: 0.85–0.96) and 1.04 (95% CI: 1.03–1.06) for positive and negative LEs, respectively. Detailed information on the crude, age- and sex-adjusted, and multivariable Poisson models is listed in Table 2 and Supplementary Tables 4, 5.

**TABLE 2:** Hypertension incidence rate ratios (IRRs) by life events (LEs) scores.

Variables	Crude models^†^	Age- and sex-adjusted models^†^	Multivariable models^†^
**The full LEs scale**			
**All LEs**			
Total score of all LEs	1.00 (0.99, 1.02)	1.02 (1.00, 1.03)*	1.02 (1.00, 1.04)*
**Positive and negative LEs^‡^**			
Score of all positive LEs	0.85 (0.79, 0.90)*	0.89 (0.84, 0.95)*	0.90 (0.85, 0.96)*
Score of all negative LEs	1.04 (1.02, 1.06)*	1.04 (1.03, 1.06)*	1.04 (1.03, 1.06)*
**Work-related LEs^§^**			
**All work-related LEs**			
Total score of work-related LEs	1.03 (0.99, 1.08)	1.07 (1.02, 1.11)*	1.08 (1.04, 1.13)*
**Positive and negative work-related LEs^‡^**			
Score of positive work-related LEs	0.84 (0.72, 0.97)*	0.87 (0.75, 1.01)	0.88 (0.75, 1.02)
Score of negative work-related LEs	1.07 (1.02, 1.12)*	1.11 (1.06, 1.16)*	1.13 (1.07, 1.18)*
**Family-related LEs ^§^**			
**All family-related LEs**			
Total score of family-related LEs	0.98 (0.95, 1.01)	1.00 (0.97, 1.03)	1.00 (0.97, 1.03)
**Positive and Negative family-related LEs^‡^**			
Score of positive family-related LEs	0.81 (0.74, 0.89)*	0.86 (0.78, 0.94)*	0.87 (0.80, 0.96)*
Score of negative family-related LEs	1.05 (1.01, 1.08)*	1.05 (1.01, 1.09)*	1.04 (1.00, 1.08)*
**Other personal LEs^§^**			
**All other personal LEs**			
Total score of other personal Les	1.03 (0.99, 1.08)	1.07 (1.03, 1.12)*	1.08 (1.03, 1.12)*
**Other positive and negative personal LEs^‡^**			
Score of other positive personal LEs	0.87 (0.76, 1.00)	0.99 (0.86, 1.14)	0.97 (0.84, 1.12)
Score of other negative personal LEs	1.07 (1.02, 1.13)*	1.09 (1.04, 1.14)*	1.10 (1.05, 1.15)*

*P < 0.05. ^†^The results are presented as IRR (95% CI). ^‡^The scores of positive and negative LEs were added concurrently into the Poisson models. ^§^Hypertension IRRs of work-related, family-related, and other personal LEs were calculated separately in different Poisson models. CI, confidence interval. Detailed information on the models is shown in Supplementary Tables 4–11.

Regarding the subcategories of LEs, the HTN IRRs in the multivariable Poisson models were 1.08 (95% CI: 1.04–1.13), 1.00 (95% CI: 0.97–1.03), and 1.08 (95% CI: 1.03–1.12) for the total score of work-related, family-related, and other personal LEs, respectively (see Table 2 and Supplementary Tables 6–8). When calculating positive and negative LEs scores separately, the HTN IRRs were 0.88 (95% CI: 0.75–1.02) and 1.13 (95% CI: 1.07–1.18) for positive and negative work-related LEs, respectively (see Table 2 and Supplementary Table 9). Regarding family-related LEs, the HTN IRRs were 0.87 (95% CI: 0.80–0.96) and 1.04 (95% CI: 1.00–1.08) for positive and negative cases, respectively (see Table 2 and Supplementary Table 10). For other personal LEs, the HTN IRRs were 0.97 (95% CI: 0.84–1.12) for positive and 1.10 (95% CI: 1.05–1.15) for negative LEs (see Table 2 and Supplementary Table 11).

### Trends Between Life Events and Hypertension Incidence Rate Ratios

**FIGURE 2:**
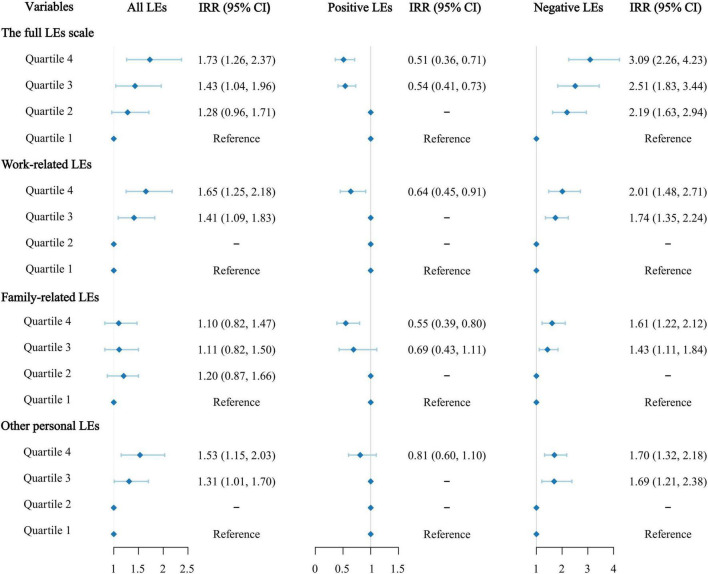
Incidence rate ratios (IRRs) and 95% confidence intervals (CIs) for hypertension by life events (LEs) scores across quartiles.

In contrast to the patients in the lowest quartile of the score of the complete LEs scale, those in quartiles two (adjusted IRR, 1.28; 95% CI, 0.96–1.71), three (adjusted IRR, 1.43; 95% CI, 1.04–1.96), and four (adjusted IRR, 1.73; 95% CI, 1.26–2.37) progressively faced an increased risk of HTN (see Figure 2 and Supplementary Table 12).

In the model that calculated positive and negative LEs scores separately, in contrast to patients in the lowest quartile of positive LEs score, participants in quartiles three (adjusted IRR, 0.54; 95% CI, 0.41–0.73) and four (adjusted IRR, 0.51; 95% CI, 0.36–0.71) progressively faced a lower risk of HTN. Meanwhile, compared with patients in the lowest quartile of negative LEs score, those in quartiles two (adjusted IRR, 2.19; 95% CI, 1.63–2.94), three (adjusted IRR, 2.51; 95% CI, 1.83–3.44) and four (adjusted IRR, 3.09; 95% CI, 2.26–4.23) progressively faced a higher risk of HTN (see Figure 2 and Supplementary Table 13). The HTN IRRs based on the quartiles of work-related, family-related, and other personal LEs scores are shown in Figure 2. The results for the other variables in the models are presented in Supplementary Tables 14–19.

**FIGURE 3:**
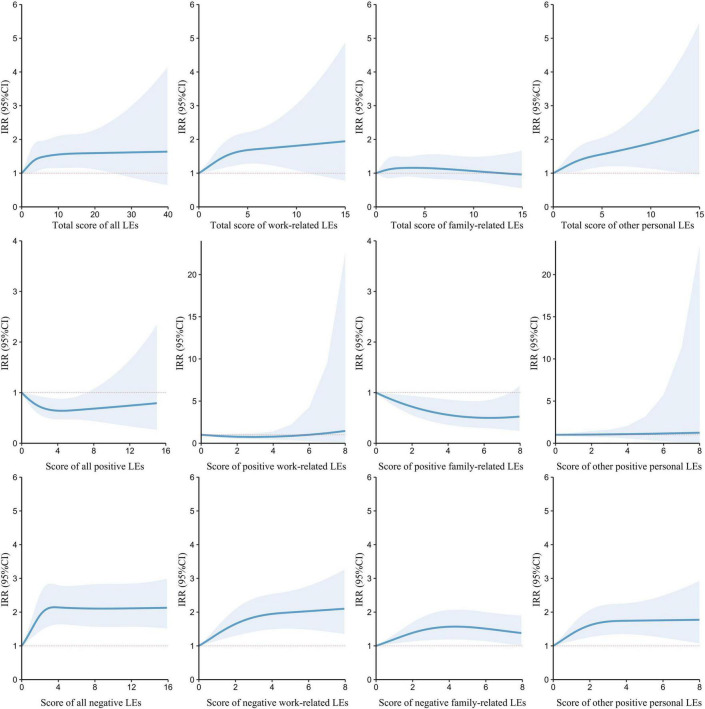
Trends between life events (LEs) scores and hypertension incidence rate ratios (IRRs) from restricted cubic spline models. The lines indicate hypertension IRRs obtained by restricted cubic spline models with five knots located at the 5th, 27.5th, 50th, 72.5th, and 95^th^ percentiles of LEs scores, and the shaded areas indicate their 95% confidence intervals (CIs).

In restricted spline curves, a non-linear association was noted between LEs and HTN risk. At first, the risk of HTN increased rapidly with the increasing LEs score (between 0 and 5 points) and then reached a plateau above 10 points. In the model that calculated positive and negative LEs scores separately, the risk of HTN decreased as the positive LEs score increased (between 0 and 4 points) and then showed an upward trend. Regarding negative LEs, the HTN risk increased rapidly as the negative LEs score increased (between 0 and 4 points) and then reached a plateau. Figure 3 shows the trends in HTN IRRs based on the scores of different LEs categories.

### Interactive, Subgroup, and Sensitivity Analyses

The association between LEs and HTN was consistent among the subgroups according to sex, education level, occupation, position title, physical activity frequency, smoking, alcohol consumption, and family history of HTN. Moreover, the association between LEs and HTN was not modified by age or BMI (see Supplementary Tables 20, 21). In the sensitivity analysis, the results were consistent with those of the main models (see Supplementary Table 22).

## Discussion

In the present study, LEs were associated with a higher HTN risk with a non-linear association. When calculating positive and negative LEs scores separately, only negative LEs were risk factors for HTN incidence while positive LEs reduced the HTN risk. The strengths of this study include a relatively large sample size, a prospective study design, and a comprehensive exploration of the dose-effect relationship. The findings of this study provide information for identifying individuals facing a high risk of HTN and advancing evidence-based approaches regarding HTN prevention among government employees.

### Comparison With Similar Studies and Interpretations

In this study, the HTN IR among government employees in Hunan Province was estimated to be 4.30% from 2018/2019 to 2019/2020, consistent with data reported previously. According to the China Health and Nutrition Survey, the HTN IRs in China increased from 1993 to 2009, which was 2.9, 3.1, 3.2, and 5.3% per person-years for the time period of 1993–2000, 1997–2004, 2000–2006, and 2004–2009, respectively (Liang et al., 2014). However, study results for HTN IRs varied across China’s different regions and eras. The IRs were reported to be 4.85% in Ningbo from 2006 to 2015 (Zheng and Mao, 2017), 3.19% in Henan Province from 2007/2008 to 2013/2014 (Chen et al., 2018), 5.48% in Beijing from 2011/2012 to 2014 (Zhang et al., 2019), and 3.68% in Anhui Province from 2014/2015 to 2016/2017 (Zhong et al., 2019). The variation in HTN IRs was attributed to diverse geographic locations, study populations, and periods, among other factors. Nevertheless, it is worth noting that the HTN IRs in China have increased substantially over the last 40 years. Thus, increased efforts are required to identify the factors associated with the risk of HTN and develop prevention strategies.

Researchers have made significant efforts to evaluate the relationship between LEs and HTN, despite inconsistent conclusions (Edwards, 1995; Sparrenberger et al., 2008; Hassoun et al., 2015). This study found that the effects of LEs on the incidence of HTN increased with LEs scores. When calculating the positive and negative LEs scores separately, only the latter was a risk factor for HTN incidence. In contrast, positive LEs were associated with a reduced risk of HTN. This finding supports and further clarifies the association between LEs and HTN.

As mentioned in previous studies, the stress induced by negative LEs can increase the risk of HTN by acting directly on relevant BP regulation systems (Sparrenberger et al., 2009; Liu M. Y. et al., 2017; Lopes, 2019). However, positive LEs act as a buffer during the onset of HTN (Caputo et al., 1998). The behavioral and physiological responses caused by LEs provide potential interpretations of the elevated HTN risk. High-risk behavioral dispositions such as poor diet, smoking, and alcohol abuse may occur after the incidence of negative LEs and contribute to the onset of HTN. Positive emotions are related to beneficial behaviors such as improved sleep quality and increased frequency of physical activity (Silton et al., 2020). Regarding physiological pathways, three main stress response systems are particularly important for analyzing the association between LEs and HTN, including the HPA axis and the autonomic nervous and immune systems (van Ockenburg et al., 2015). Stress that is induced by negative LEs can activate the HPA axis and participate in the control of whole-body homeostasis (Chida and Hamer, 2008). The autonomic nervous system mediates stress responses via the SNS and PSNS (Chida and Hamer, 2008). SNS stimulation causes vasoconstriction and increases BP. The PSNS mediates stress responses through vagal and sacral parasympathetic efferents and influence BP regulation. Regarding the immune system, evidence shows that SNS and angiotensin hormones can stimulate the release of interleukin-6 (März et al., 1998; Bürger et al., 2001), which can cause or facilitate vasoconstriction (Steptoe et al., 2007). Conversely, the positive emotions inspired by positive LEs are related to lower cortisol levels and reduced inflammation (Steptoe et al., 2005; Sin et al., 2015), and can buffer the response to negative stimuli (Silton et al., 2020).

In this study, the dose-effect association between LEs and HTN reached a plateau following an upward trend. There are several possible explanations for this phenomenon. First, the adaptive changes caused by one LE may enable organisms to maintain homeostasis in response to the adverse effects of other LEs (Chauhan et al., 2015). Second, people may begin seeking support from their families and friends after experiencing a negative LE. The ensuing increased social support, per the stress-buffering thesis, can moderate the association between stress and health outcomes (Bowen et al., 2014; Miao Jonasson et al., 2020). Third, people who have experienced LEs can learn from their experiences, thus improving their coping strategies toward LEs (van den Heuvel et al., 2020). Finally, some individuals who experienced LEs may suffer from affective dysregulation or somatic symptoms and seek professional help, preventing the onset of HTN to a certain extent (Beards et al., 2020; Schneider et al., 2021).

Notably, not all LEs domains are equally associated with the onset of HTN. Regarding different subdomains of LEs, it was found that the total score of work-related LEs, personal LEs, and all subcategories of negative LEs were associated significantly with an increased risk of HTN. However, among positive LEs, only family-related LEs scores were associated with a lower risk. Family-related positive LEs might be accompanied by an increased sense of belonging and cohesion as well as support from family (Black and Lobo, 2008; Kiecolt-Glaser and Wilson, 2017). This plays a protective role in health conditions and forms a buffer against the influence of negative LEs (Child et al., 2022). This may also contribute to the stronger protective effect of positive family-related LEs compared with work-related and other personal cases in this study. Moreover, positive work-related LEs may be accompanied by increased work tasks, resulting in less time for exercise and relaxation, which has adverse impacts on health (Liu X. et al., 2017). Researchers in other countries have reported discrepant results regarding the effects of different categories of LEs (Odia and Uzogara, 1988; Sparrenberger et al., 2008). These discrepancies may result from cultural and political diversity. Thus, findings from this study could help researchers improve their understanding of negative and positive LEs from work, family, and other personal matters and their association with HTN in the Chinese cultural context.

### Implications for Prevention and Intervention

The findings from this study have several implications. First, these findings provide evidence regarding the non-linear relationship between LEs and HTN among Chinese government employees. Second, the finding emphasize the importance of interventions for individuals who experienced negative LEs. Physicians, family members, and friends can help those individuals reduce the risk of HTN by providing social support, exposure to appropriate coping strategies, and supervising their health-related behaviors. Third, managers could consider providing mental health services to employees, which can not only benefit their psychological wellbeing and improve productivity but also prevent HTN, possible cardiovascular diseases, and its long-term associated mortality. Finally, policymakers should further highlight the importance of LEs and associated psychosocial stress in the etiology of HTN and take them into consideration in the development of HTN prevention strategies and initiatives.

### Limitations and Future Research

This study has several limitations. First, the follow-up rate was lower than expected, and baseline characteristics were not balanced between the excluded and included populations. The low follow-up rate might have been due to the tedious follow-up process, and it was unclear whether this imbalance impacted the results. For future studies, the workflow has to be optimized, lost participants need to be traced, and findings need to be verified further. Second, only the LEs that occurred in the previous year were studied. However, the risk of HTN due to long-term LEs is still not clear. In future studies, the scale must be updated to include LEs that occur during different periods of an individual’s lifetime. Third, some of the potentially influential factors, such as personal traits and social support, were not considered in the present study. Moreover, the mediating effects of some variables were not considered. In future research, these variables should be included, along with the mediator effect. Finally, the follow-up period of this study was only 1 year. And the exact time points for the occurrence of LEs and the onset of HTN were not clear. Thus, the time differences among participants, which could influence the risk of LEs, were not considered in this study. We will continue the follow-up to explore the long-term effects of LEs and consider the time variable in future studies.

Further research is crucial to gain in-depth understanding of the relationship between LEs and HTN incidence. Further laboratory experiments and psychophysiological studies are required to elucidate the underlying mechanisms. In addition, well-designed prospective epidemiological studies with mediation analysis represent another important avenue for mechanistic exploration. Intervention trials are also essential for piloting and evaluating preventive measures and strategies.

## Conclusion

LEs displayed a non-linear association with an increased risk of HTN. When calculating the positive and negative LEs scores separately, only the latter was associated with an increased risk of HTN, whereas positive LEs reduced the risk. Regarding the subcategories of LEs, the negative LEs scores across all categories were associated significantly with an increased risk of HTN. However, only family-related LEs score among the positive LEs was associated with a lower risk of HTN. These findings have important implications for the prevention, early identification, and management of HTN, which are essential for reducing the burden associated with HTN.

## Data Availability Statement

The data that support the findings of this study are available on request from the corresponding author.

## Ethics Statement

The studies involving human participants were reviewed and approved by the Institutional Review Board of Xiangya School of Public Health, Central South University. The patients/participants provided their written informed consent to participate in this study.

## Author Contributions

FO and SX: conceptualization. FO and JH: formal analysis and writing–original draft preparation. FO, JH, DQ, and DL: investigation. DQ and FO: data management. LL, JB, and XC: writing–review and editing. DL and YD: project administration. All authors read and approved the final manuscript.

## Conflict of Interest

The authors declare that the research was conducted in the absence of any commercial or financial relationships that could be construed as a potential conflict of interest.

## Publisher’s Note

All claims expressed in this article are solely those of the authors and do not necessarily represent those of their affiliated organizations, or those of the publisher, the editors and the reviewers. Any product that may be evaluated in this article, or claim that may be made by its manufacturer, is not guaranteed or endorsed by the publisher.
