# Supplementary material for: The Association Between Life Events and Incidence of Hypertension Among Government Employees in China: A Prospective Cohort Study
Source: Front Psychol. 2022 May 30;13:822610. doi: 10.3389/fpsyg.2022.822610 (PMC9190203; doi:10.3389/fpsyg.2022.822610)
Supplement: Supplementary file 1 [file Data_Sheet_1.docx]

Supplementary Material

# 1. Supplemental Table

## **Table S1. Life Events Scale**

| **Life Events Scale** | |
| --- | --- |
| Q1 In the past year, have the following event occurred: being in love or engaged? | |
| A. Yes B. No | |
|  | Q1.1 How much stress has this event inspired to you? |
|  | A. Mild B. Moderate C. Heavy D. Extremely heavy |
| Q2 In the past year, have the following event occurred: a relationship failed or broken up? | |
| A. Yes B. No | |
|  | Q2.1 How much stress has this event inspired to you? |
|  | A. Mild B. Moderate C. Heavy D. Extremely heavy |
| Q3 In the past year, have the following event occurred: getting married | |
| A. Yes B. No | |
|  | Q3.1 How much stress has this event inspired to you? |
|  | A. Mild B. Moderate C. Heavy D. Extremely heavy |
| Q4 In the past year, have the following event occurred: pregnant by yourself or your spouse? | |
| A. Yes B. No | |
|  | Q4.1 How much stress has this event inspired to you? |
|  | A. Mild B. Moderate C. Heavy D. Extremely heavy |
| Q5 In the past year, have the following event occurred: abortion of yourself or your spouse? | |
| A. Yes B. No | |
|  | Q5.1 How much stress has this event inspired to you? |
|  | A. Mild B. Moderate C. Heavy D. Extremely heavy |
| Q6 In the past year, have the following event occurred: a new addition to the family? | |
| A. Yes B. No | |
|  | Q6.1 How much stress has this event inspired to you? |
|  | A. Mild B. Moderate C. Heavy D. Extremely heavy |
| Q7 In the past year, have the following event occurred: having a disagreement with a loved one or parents? | |
| A. Yes B. No | |
|  | Q7.1 How much stress has this event inspired to you? |
|  | A. Mild B. Moderate C. Heavy D. Extremely heavy |
| Q8 In the past year, have the following event occurred: having bad relationship with your spouse? | |
| A. Yes B. No | |
|  | Q8.1 How much stress has this event inspired to you? |
|  | A. Mild B. Moderate C. Heavy D. Extremely heavy |
| Q9 In the past year, have the following event occurred: being divided with your spouse by discord? | |
| A. Yes B. No | |
|  | Q9.1 How much stress has this event inspired to you? |
|  | A. Mild B. Moderate C. Heavy D. Extremely heavy |
| Q10 In the past year, have the following event occurred: sexual dissatisfaction? | |
| A. Yes B. No | |
|  | Q10.1 How much stress has this event inspired to you? |
|  | A. Mild B. Moderate C. Heavy D. Extremely heavy |
| Q11 In the past year, have the following event occurred: separation with your spouse because of work? | |
| A. Yes B. No | |
|  | Q11.1 How much stress has this event inspired to you? |
|  | A. Mild B. Moderate C. Heavy D. Extremely heavy |
| Q12 In the past year, have the following event occurred: you or your spouse having an affair? | |
| A. Yes B. No | |
|  | Q12.1 How much stress has this event inspired to you? |
|  | A. Mild B. Moderate C. Heavy D. Extremely heavy |
| Q13 In the past year, have the following event occurred: you and your spouse being reconciled? | |
| A. Yes B. No | |
|  | Q13.1 How much stress has this event inspired to you? |
|  | A. Mild B. Moderate C. Heavy D. Extremely heavy |
| Q14 In the past year, have the following event occurred: over-index fertility? | |
| A. Yes B. No | |
|  | Q14.1 How much stress has this event inspired to you? |
|  | A. Mild B. Moderate C. Heavy D. Extremely heavy |
| Q15 In the past year, have the following event occurred: sterilization of yourself or your spouse? | |
| A. Yes B. No | |
|  | Q15.1 How much stress has this event inspired to you? |
|  | A. Mild B. Moderate C. Heavy D. Extremely heavy |
| Q16 In the past year, have the following event occurred: death of your spouse? | |
| A. Yes B. No | |
|  | Q16.1 How much stress has this event inspired to you? |
|  | A. Mild B. Moderate C. Heavy D. Extremely heavy |
| Q17 In the past year, have the following event occurred: divorce? | |
| A. Yes B. No | |
|  | Q17.1 How much stress has this event inspired to you? |
|  | A. Mild B. Moderate C. Heavy D. Extremely heavy |
| Q18 In the past year, have the following event occurred: children failing in enter a higher school or getting a job? | |
| A. Yes B. No | |
|  | Q18.1 How much stress has this event inspired to you? |
|  | A. Mild B. Moderate C. Heavy D. Extremely heavy |
| Q19 In the past year, have the following event occurred: struggling in children discipline? | |
| A. Yes B. No | |
|  | Q19.1 How much stress has this event inspired to you? |
|  | A. Mild B. Moderate C. Heavy D. Extremely heavy |
| Q20 In the past year, have the following event occurred: long-term absence of children? | |
| A. Yes B. No | |
|  | Q20.1 How much stress has this event inspired to you? |
|  | A. Mild B. Moderate C. Heavy D. Extremely heavy |
| Q21 In the past year, have the following event occurred: parents not getting along well? | |
| A. Yes B. No | |
|  | Q21.1 How much stress has this event inspired to you? |
|  | A. Mild B. Moderate C. Heavy D. Extremely heavy |
| Q22 In the past year, have the following event occurred: meeting family financial difficulties? | |
| A. Yes B. No | |
|  | Q22.1 How much stress has this event inspired to you? |
|  | A. Mild B. Moderate C. Heavy D. Extremely heavy |
| Q23 In the past year, have the following event occurred: being in debt? | |
| A. Yes B. No | |
|  | Q23.1 How much stress has this event inspired to you? |
|  | A. Mild B. Moderate C. Heavy D. Extremely heavy |
| Q24 In the past year, have the following event occurred: improving economic situation markedly? | |
| A. Yes B. No | |
|  | Q24.1 How much stress has this event inspired to you? |
|  | A. Mild B. Moderate C. Heavy D. Extremely heavy |
| Q25 In the past year, have the following event occurred: family members seriously ill or seriously injured? | |
| A. Yes B. No | |
|  | Q25.1 How much stress has this event inspired to you? |
|  | A. Mild B. Moderate C. Heavy D. Extremely heavy |
| Q26 In the past year, have the following event occurred: Death of a family member? | |
| A. Yes B. No | |
|  | Q26.1 How much stress has this event inspired to you? |
|  | A. Mild B. Moderate C. Heavy D. Extremely heavy |
| Q27 In the past year, have the following event occurred: seriously ill or seriously injured by yourself? | |
| A. Yes B. No | |
|  | Q27.1 How much stress has this event inspired to you? |
|  | A. Mild B. Moderate C. Heavy D. Extremely heavy |
| Q28 In the past year, have the following event occurred: housing shortage? | |
| A. Yes B. No | |
|  | Q28.1 How much stress has this event inspired to you? |
|  | A. Mild B. Moderate C. Heavy D. Extremely heavy |
| Q29 In the past year, have the following event occurred: being unemployed? | |
| A. Yes B. No | |
|  | Q29.1 How much stress has this event inspired to you? |
|  | A. Mild B. Moderate C. Heavy D. Extremely heavy |
| Q30 In the past year, have the following event occurred: taking up an occupation? | |
| A. Yes B. No | |
|  | Q30.1 How much stress has this event inspired to you? |
|  | A. Mild B. Moderate C. Heavy D. Extremely heavy |
| Q31 In the past year, have the following event occurred: failing in the college entrance examination? | |
| A. Yes B. No | |
|  | Q31.1 How much stress has this event inspired to you? |
|  | A. Mild B. Moderate C. Heavy D. Extremely heavy |
| Q32 In the past year, have the following event occurred: withholding bonus or penalty? | |
| A. Yes B. No | |
|  | Q32.1 How much stress has this event inspired to you? |
|  | A. Mild B. Moderate C. Heavy D. Extremely heavy |
| Q33 In the past year, have the following event occurred: outstanding personal achievements? | |
| A. Yes B. No | |
|  | Q33.1 How much stress has this event inspired to you? |
|  | A. Mild B. Moderate C. Heavy D. Extremely heavy |
| Q34 In the past year, have the following event occurred: getting promotion? | |
| A. Yes B. No | |
|  | Q34.1 How much stress has this event inspired to you? |
|  | A. Mild B. Moderate C. Heavy D. Extremely heavy |
| Q35 In the past year, have the following event occurred: dissatisfaction with the present job? | |
| A. Yes B. No | |
|  | Q35.1 How much stress has this event inspired to you? |
|  | A. Mild B. Moderate C. Heavy D. Extremely heavy |
| Q36 In the past year, have the following event occurred: feeling stressful in study or work? | |
| A. Yes B. No | |
|  | Q36.1 How much stress has this event inspired to you? |
|  | A. Mild B. Moderate C. Heavy D. Extremely heavy |
| Q37 In the past year, have the following event occurred: tension with superiors? | |
| A. Yes B. No | |
|  | Q37.1 How much stress has this event inspired to you? |
|  | A. Mild B. Moderate C. Heavy D. Extremely heavy |
| Q38 In the past year, have the following event occurred: disharmony with colleagues | |
| A. Yes B. No | |
|  | Q38.1 How much stress has this event inspired to you? |
|  | A. Mild B. Moderate C. Heavy D. Extremely heavy |
| Q39 In the past year, have the following event occurred: the first time away from a foreign country? | |
| A. Yes B. No | |
|  | Q39.1 How much stress has this event inspired to you? |
|  | A. Mild B. Moderate C. Heavy D. Extremely heavy |
| Q40 In the past year, have the following event occurred: major changes in lifestyle (e.g., diet or sleep)? | |
| A. Yes B. No | |
|  | Q40.1 How much stress has this event inspired to you? |
|  | A. Mild B. Moderate C. Heavy D. Extremely heavy |
| Q41 In the past year, have the following event occurred: being retired or having no specific job arranged? | |
| A. Yes B. No | |
|  | Q41.1 How much stress has this event inspired to you? |
|  | A. Mild B. Moderate C. Heavy D. Extremely heavy |
| Q42 In the past year, have the following event occurred: a good friend's seriously injured or ill | |
| A. Yes B. No | |
|  | Q42.1 How much stress has this event inspired to you? |
|  | A. Mild B. Moderate C. Heavy D. Extremely heavy |
| Q43 In the past year, have the following event occurred: death of a friend | |
| A. Yes B. No | |
|  | Q43.1 How much stress has this event inspired to you? |
|  | A. Mild B. Moderate C. Heavy D. Extremely heavy |
| Q44 In the past year, have the following event occurred: being misunderstood, wrongly blamed, falsely accused? | |
| A. Yes B. No | |
|  | Q44.1 How much stress has this event inspired to you? |
|  | A. Mild B. Moderate C. Heavy D. Extremely heavy |
| Q45 In the past year, have the following event occurred: getting involved in civil legal disputes? | |
| A. Yes B. No | |
|  | Q45.1 How much stress has this event inspired to you? |
|  | A. Mild B. Moderate C. Heavy D. Extremely heavy |
| Q46 In the past year, have the following event occurred: being detained, tried? | |
| A. Yes B. No | |
|  | Q46.1 How much stress has this event inspired to you? |
|  | A. Mild B. Moderate C. Heavy D. Extremely heavy |
| Q47 In the past year, have the following event occurred: suffering from loss by theft? | |
| A. Yes B. No | |
|  | Q47.1 How much stress has this event inspired to you? |
|  | A. Mild B. Moderate C. Heavy D. Extremely heavy |
| Q48 In the past year, have the following event occurred: suffering from scare, accident or natural disaster? | |
| A. Yes B. No | |
|  | Q48.1 How much stress has this event inspired to you? |
|  | A. Mild B. Moderate C. Heavy D. Extremely heavy |

# 2. Supplemental Table

## **Table S2. Classification of life events**

| **Category** | **Subcategory** | **Item number*** |
| --- | --- | --- |
| Work-Related life events | Positive | Q33 / Q34 |
|  | Negative | Q29 / Q30 / Q31 / Q32 / Q35 / Q35 / Q37 / Q38 / Q39 |
| Family-Related life events | Positive | Q3/ Q4 / Q6 / Q13 |
|  | Negative | Q5 / Q7 / Q8 / Q9 / Q10 / Q11 / Q12 / Q14 / Q15 / Q16 / Q17 / Q18 / Q19 / Q20 / Q21 / Q22 / Q25 / Q26 / Q41 |
| Other personal life events | Positive | Q1/ Q 24 |
|  | Negative | Q2/ Q23 / Q27 / Q28 / Q40 / Q42 / Q43 / Q44 / Q45 / Q46 / Q47 / Q48 |

*The numbers refer to the item numbers in the life events scale (Table S1).

# 3. Supplemental Table

## **Table S3. The comparison of the excluded and included population**

| **Variables** | **Overall population** | **Excluded population** | **Included population** | ***P* value** |
| --- | --- | --- | --- | --- |
| Number of participants | 11433 | 3356 | 8077 |  |
| Age (year) | 36.0 (30.3, 44.6) | 37.0 (30.0, 47.0) | 35.7 (30.5, 43.4) | <0.001 |
| BMI (kg/m^2^) | 22.7 (20.6, 25.1) | 23.2 (20.9, 25.8) | 22.5 (20.7, 24.8) | <0.001 |
| Baseline SBP (mmHg) | 116.0 (107.0, 125.0) | 120.0 (110.0, 131.0) | 114.0 (107.0, 123.0) | <0.001 |
| Baseline DBP (mmHg) | 70.0 (64.0, 78.0) | 74.0 (66.0, 83.0) | 69.0 (63.0, 76.0) | <0.001 |
| **Sex** |  |  |  |  |
| Male | 4011 (35.1) | 1442 (43.0) | 2569 (31.8) | <0.001 |
| Female | 7422 (64.9) | 1914 (57.0) | 5508 (68.2) |  |
| **Marital status** |  |  |  |  |
| Unmarried | 2141 (18.7) | 603 (18.0) | 1538 (19.0) | 0.035 |
| Married | 9017 (78.9) | 2655 (79.1) | 6362 (78.8) |  |
| Divorced or widowed | 275 (2.4) | 98 (2.9) | 177 (2.2) |  |
| **Educational level** |  |  |  |  |
| High school or below | 562 (4.9) | 312 (9.3) | 250 (3.1) | <0.001 |
| Bachelor | 7197 (62.9) | 2131 (63.5) | 5066 (62.7) |  |
| Postgraduate or above | 3674 (32.1) | 913 (27.2) | 2761 (34.2) |  |
| **Occupation** |  |  |  |  |
| Government officials | 509 (4.5) | 235 (7.0) | 274 (3.4) | <0.001 |
| Teachers or researchers | 2427 (21.2) | 755 (22.5) | 1672 (20.7) |  |
| Police | 434 (3.8) | 93 (2.8) | 341 (4.2) |  |
| Medical staff | 7008 (61.3) | 1973 (58.8) | 5035 (62.3) |  |
| Other governmental employees | 1055 (9.2) | 300 (8.9) | 755 (9.3) |  |
| **Position title** |  |  |  |  |
| Junior professional staff | 5385 (47.1) | 1571 (46.8) | 3814 (47.2) | <0.001 |
| Intermediate professional  staff | 4077 (35.7) | 1095 (32.6) | 2982 (36.9) |  |
| Senior professional staff | 1971 (17.2) | 690 (20.6) | 1281 (15.9) |  |
| **Physical activity** |  |  |  |  |
| <1/week | 5467 (47.8) | 1602 (47.7) | 3865 (47.9) | 0.971 |
| 1-2/week | 3020 (26.4) | 895 (26.7) | 2125 (26.3) |  |
| >3/week | 2946 (25.8) | 859 (25.6) | 2087 (25.8) |  |
| **Smoking** |  |  |  |  |
| Nonsmokers | 10089 (88.2) | 2835 (84.5) | 7254 (89.8) | <0.001 |
| Current Smokers | 1180 (10.3) | 457 (13.6) | 723 (9.0) |  |
| Former smokers | 164 (1.4) | 64 (1.9) | 100 (1.2) |  |
| **Alcohol consumption** |  |  |  |  |
| Non-drinkers | 9506 (83.1) | 2700 (80.5) | 6806 (84.3) | <0.001 |
| Current drinkers | 1850 (16.2) | 631 (18.8) | 1219 (15.1) |  |
| Former drinkers | 77 (0.7) | 25 (0.7) | 52 (0.6) |  |
| **Family history of HT** |  |  |  |  |
| With family history of HT | 7523 (65.8) | 2115 (63.0) | 5408 (67.0) | <0.001 |
| Without family history of HT | 3910 (34.2) | 1241 (37.0) | 2669 (33.0) |  |

*Note:* HT, hypertension. SBP, systolic blood pressure; DBP, diastolic blood pressure; BMI, body mass index; kg, kilogram; m, meter.

# 4. Supplemental Table

## **Table S4. Incidence rate ratios of hypertension by** **total score of all life events**

| **Variable** | **Estimate** | **SE** | ***P*** | **IRR** | **95% CI** | |
| --- | --- | --- | --- | --- | --- | --- |
|  |  |  |  |  | **Lower** | **Upper** |
| Crude Poisson model |  |  |  |  |  |  |
| Intercept | -3.15 | 0.07 | <0.001 | 0.04 | 0.04 | 0.05 |
| Total score of all life events | 0.00 | 0.01 | 0.858 | 1.00 | 0.99 | 1.02 |
| Age- and sex-adjusted Poisson model |  |  |  |  |  |  |
| Intercept | -4.57 | 0.28 | <0.001 | 0.01 | 0.01 | 0.02 |
| Age | 0.04 | 0.01 | <0.001 | 1.04 | 1.03 | 1.06 |
| **Sex** |  |  |  |  |  |  |
| Male | Reference |  |  |  |  |  |
| Female | -0.63 | 0.11 | <0.001 | 0.53 | 0.43 | 0.66 |
| Total score of all life events | 0.02 | 0.01 | 0.029 | 1.02 | 1.00 | 1.03 |
| Multivariable Poisson model |  |  |  |  |  |  |
| Intercept | -16.10 | 0.95 | <0.001 | 0.00 | 0.00 | 0.00 |
| Age | 0.04 | 0.01 | <0.001 | 1.04 | 1.02 | 1.05 |
| **Sex** |  |  |  |  |  |  |
| Male | Reference |  |  |  |  |  |
| Female | -0.02 | 0.14 | 0.885 | 0.98 | 0.74 | 1.30 |
| BMI | 0.01 | 0.01 | 0.416 | 1.01 | 0.99 | 1.02 |
| SBP | 0.08 | 0.01 | <0.001 | 1.08 | 1.06 | 1.10 |
| DBP | 0.04 | 0.01 | <0.001 | 1.04 | 1.02 | 1.06 |
| Total score of all life events | 0.02 | 0.01 | 0.016 | 1.02 | 1.00 | 1.04 |
| **Marital status** |  |  |  |  |  |  |
| Single | Reference |  |  |  |  |  |
| Married | -0.12 | 0.18 | 0.501 | 0.88 | 0.62 | 1.27 |
| Widowed or divorced | 0.18 | 0.35 | 0.615 | 1.19 | 0.60 | 2.37 |
| **Educational level** |  |  |  |  |  |  |
| High school or below | Reference |  |  |  |  |  |
| Bachelor | -0.45 | 0.22 | 0.044 | 0.64 | 0.41 | 0.99 |
| Postgraduate or above | -0.77 | 0.26 | 0.003 | 0.46 | 0.28 | 0.77 |
| **Occupation** |  |  |  |  |  |  |
| Government officials | Reference |  |  |  |  |  |
| Other governmental employees | -0.33 | 0.29 | 0.243 | 0.72 | 0.41 | 1.25 |
| Teachers or researchers | -0.18 | 0.25 | 0.476 | 0.83 | 0.51 | 1.37 |
| Police | -0.21 | 0.30 | 0.485 | 0.81 | 0.45 | 1.46 |
| Medical staff | -0.24 | 0.24 | 0.314 | 0.79 | 0.49 | 1.26 |
| **Position title** |  |  |  |  |  |  |
| Junior professional staff | Reference |  |  |  |  |  |
| Intermediate professional staff | -0.01 | 0.14 | 0.918 | 0.99 | 0.75 | 1.30 |
| Senior professional staff | -0.24 | 0.20 | 0.223 | 0.79 | 0.53 | 1.16 |
| **Physical activity** |  |  |  |  |  |  |
| <1/week | Reference |  |  |  |  |  |
| 1-2/week | -0.32 | 0.14 | 0.021 | 0.73 | 0.55 | 0.95 |
| >3/week | -0.47 | 0.14 | <0.001 | 0.62 | 0.48 | 0.81 |
| **Smoking** |  |  |  |  |  |  |
| Nonsmokers | Reference |  |  |  |  |  |
| Current Smokers | -0.31 | 0.18 | 0.083 | 0.74 | 0.52 | 1.04 |
| Former smokers | -0.21 | 0.39 | 0.597 | 0.81 | 0.38 | 1.75 |
| **Alcohol consumption** |  |  |  |  |  |  |
| Non-drinkers | Reference |  |  |  |  |  |
| Current drinkers | 0.36 | 0.14 | 0.011 | 1.43 | 1.09 | 1.88 |
| Former drinkers | -1.57 | 1.01 | 0.119 | 0.21 | 0.03 | 1.50 |
| **Family history of hypertension** |  |  |  |  |  |  |
| With family history of hypertension | Reference |  |  |  |  |  |
| Without family history of hypertension | 0.24 | 0.11 | 0.036 | 1.27 | 1.02 | 1.58 |

*Note*: SBP, systolic blood pressure; DBP, diastolic blood pressure; BMI, body mass index; IRR, incidence rate ratios; CI, confidence interval; SE=standard error.

# 5. Supplemental Table

## **Table S5. Incidence rate ratios of hypertension by positive and negative life events**

| **Variable** | **Estimate** | **SE** | ***P*** | **IRR** | **95% CI** | |
| --- | --- | --- | --- | --- | --- | --- |
|  |  |  |  |  | **Lower** | **Upper** |
| Crude Poisson model |  |  |  |  |  |  |
| Intercept | -3.08 | 0.07 | <0.001 | 0.05 | 0.04 | 0.05 |
| Score of all positive life events | -0.17 | 0.03 | <0.001 | 0.85 | 0.79 | 0.90 |
| Score of all negative life events | 0.04 | 0.01 | <0.001 | 1.04 | 1.02 | 1.06 |
| Age- and sex-adjusted Poisson model |  |  |  |  |  |  |
| Intercept | -4.21 | 0.28 | <0.001 | 0.01 | 0.01 | 0.03 |
| Age | 0.04 | 0.01 | <0.001 | 1.04 | 1.03 | 1.05 |
| **Sex** |  |  |  |  |  |  |
| Male | Reference |  |  |  |  |  |
| Female | -0.65 | 0.11 | <0.001 | 0.52 | 0.42 | 0.65 |
| Score of all positive life events | -0.12 | 0.03 | <0.001 | 0.89 | 0.84 | 0.95 |
| Score of all negative life events | 0.04 | 0.01 | <0.001 | 1.04 | 1.03 | 1.06 |
| Multivariable Poisson model |  |  |  |  |  |  |
| Intercept | -15.85 | 0.94 | <0.001 | 0.00 | 0.00 | 0.00 |
| Age | 0.03 | 0.01 | 0.001 | 1.03 | 1.01 | 1.05 |
| **Sex** |  |  |  |  |  |  |
| Male | Reference |  |  |  |  |  |
| Female | -0.03 | 0.14 | 0.849 | 0.97 | 0.73 | 1.29 |
| BMI | 0.01 | 0.01 | 0.431 | 1.01 | 0.99 | 1.02 |
| SBP | 0.08 | 0.01 | <0.001 | 1.08 | 1.07 | 1.10 |
| DBP | 0.04 | 0.01 | <0.001 | 1.04 | 1.02 | 1.06 |
| Score of all positive life events | -0.10 | 0.03 | 0.001 | 0.90 | 0.85 | 0.96 |
| Score of all negative life events | 0.04 | 0.01 | <0.001 | 1.04 | 1.03 | 1.06 |
| **Marital status** |  |  |  |  |  |  |
| Single | Reference |  |  |  |  |  |
| Married | -0.07 | 0.19 | 0.722 | 0.94 | 0.65 | 1.35 |
| Widowed or divorced | 0.06 | 0.35 | 0.857 | 1.07 | 0.53 | 2.14 |
| **Educational level** |  |  |  |  |  |  |
| High school or below | Reference |  |  |  |  |  |
| Bachelor | -0.46 | 0.22 | 0.038 | 0.63 | 0.41 | 0.97 |
| Postgraduate or above | -0.73 | 0.26 | 0.004 | 0.48 | 0.29 | 0.79 |
| **Occupation** |  |  |  |  |  |  |
| Government officials | Reference |  |  |  |  |  |
| Other governmental employees | -0.33 | 0.29 | 0.251 | 0.72 | 0.41 | 1.26 |
| Teachers or researchers | -0.16 | 0.25 | 0.522 | 0.85 | 0.52 | 1.40 |
| Police | -0.24 | 0.30 | 0.429 | 0.79 | 0.44 | 1.42 |
| Medical staff | -0.23 | 0.24 | 0.343 | 0.80 | 0.50 | 1.27 |
| **Position title** |  |  |  |  |  |  |
| Junior professional staff | Reference |  |  |  |  |  |
| Intermediate professional staff | -0.03 | 0.14 | 0.856 | 0.97 | 0.74 | 1.29 |
| Senior professional staff | -0.26 | 0.20 | 0.198 | 0.77 | 0.53 | 1.14 |
| **Physical activity** |  |  |  |  |  |  |
| <1/week | Reference |  |  |  |  |  |
| 1-2/week | -0.32 | 0.14 | 0.022 | 0.73 | 0.55 | 0.95 |
| >3/week | -0.47 | 0.14 | 0.001 | 0.62 | 0.48 | 0.81 |
| **Smoking** |  |  |  |  |  |  |
| Nonsmokers | Reference |  |  |  |  |  |
| Current Smokers | -0.34 | 0.18 | 0.055 | 0.71 | 0.50 | 1.01 |
| Former smokers | -0.24 | 0.39 | 0.536 | 0.78 | 0.36 | 1.69 |
| **Alcohol consumption** |  |  |  |  |  |  |
| Non-drinkers | Reference |  |  |  |  |  |
| Current drinkers | 0.38 | 0.14 | 0.007 | 1.46 | 1.11 | 1.92 |
| Former drinkers | -1.59 | 1.01 | 0.114 | 0.20 | 0.03 | 1.47 |
| **Family history of hypertension** |  |  |  |  |  |  |
| With family history of hypertension | Reference |  |  |  |  |  |
| Without family history of hypertension | 0.22 | 0.11 | 0.046 | 1.25 | 1.00 | 1.56 |

*Note*: SBP, systolic blood pressure; DBP, diastolic blood pressure; BMI, body mass index; IRR, incidence rate ratios; CI, confidence interval; SE=standard error.

# 6. Supplemental Table

## **Table S6. Incidence rate ratios of hypertension by total score of work-related life events**

| **Variable** | **Estimate** | **SE** | **P** | **IRR** | **95% CI** | |
| --- | --- | --- | --- | --- | --- | --- |
|  |  |  |  |  | **Lower** | **Upper** |
| Crude Poisson model |  |  |  |  |  |  |
| Intercept | -3.18 | 0.06 | <0.001 | 0.04 | 0.04 | 0.05 |
| Total score of work-related life events | 0.03 | 0.02 | 0.151 | 1.03 | 0.99 | 1.08 |
| Age- and sex-adjusted Poisson model |  |  |  |  |  |  |
| Intercept | -4.60 | 0.27 | <0.001 | 0.01 | 0.01 | 0.02 |
| Age | 0.04 | 0.01 | <0.001 | 1.05 | 1.03 | 1.06 |
| **Sex** |  |  |  |  |  |  |
| Male | Reference |  |  |  |  |  |
| Female | -0.62 | 0.11 | <0.001 | 0.54 | 0.43 | 0.67 |
| Total score of work-related life events | 0.06 | 0.02 | 0.003 | 1.07 | 1.02 | 1.11 |
| Multivariable Poisson model |  |  |  |  |  |  |
| Intercept | -16.22 | 0.95 | <0.001 | 0.00 | 0.00 | 0.00 |
| Age | 0.04 | 0.01 | <0.001 | 1.04 | 1.02 | 1.05 |
| **Sex** |  |  |  |  |  |  |
| Male | Reference |  |  |  |  |  |
| Female | -0.03 | 0.14 | 0.849 | 0.97 | 0.73 | 1.29 |
| BMI | 0.01 | 0.01 | 0.423 | 1.01 | 0.99 | 1.02 |
| SBP | 0.08 | 0.01 | <0.001 | 1.08 | 1.06 | 1.10 |
| DBP | 0.04 | 0.01 | <0.001 | 1.04 | 1.02 | 1.06 |
| Total score of work-related life events | 0.08 | 0.02 | <0.001 | 1.08 | 1.04 | 1.13 |
| **Marital status** |  |  |  |  |  |  |
| Single | Reference |  |  |  |  |  |
| Married | -0.06 | 0.18 | 0.736 | 0.94 | 0.66 | 1.35 |
| Widowed or divorced | 0.26 | 0.35 | 0.460 | 1.29 | 0.66 | 2.55 |
| **Educational level** |  |  |  |  |  |  |
| High school or below | Reference |  |  |  |  |  |
| Bachelor | -0.45 | 0.22 | 0.043 | 0.64 | 0.41 | 0.99 |
| Postgraduate or above | -0.79 | 0.26 | 0.002 | 0.46 | 0.28 | 0.75 |
| **Occupation** |  |  |  |  |  |  |
| Government officials | Reference |  |  |  |  |  |
| Other governmental employees | -0.30 | 0.29 | 0.301 | 0.74 | 0.42 | 1.30 |
| Teachers or researchers | -0.16 | 0.25 | 0.537 | 0.85 | 0.52 | 1.41 |
| Police | -0.19 | 0.30 | 0.523 | 0.83 | 0.46 | 1.49 |
| Medical staff | -0.20 | 0.24 | 0.405 | 0.82 | 0.51 | 1.31 |
| **Position title** |  |  |  |  |  |  |
| Junior professional staff | Reference |  |  |  |  |  |
| Intermediate professional staff | -0.03 | 0.14 | 0.846 | 0.97 | 0.74 | 1.28 |
| Senior professional staff | -0.27 | 0.20 | 0.169 | 0.76 | 0.52 | 1.12 |
| **Physical activity** |  |  |  |  |  |  |
| <1/week | Reference |  |  |  |  |  |
| 1-2/week | -0.32 | 0.14 | 0.022 | 0.73 | 0.55 | 0.96 |
| >3/week | -0.46 | 0.14 | 0.001 | 0.63 | 0.48 | 0.82 |
| **Smoking** |  |  |  |  |  |  |
| Nonsmokers | Reference |  |  |  |  |  |
| Current Smokers | -0.32 | 0.18 | 0.070 | 0.73 | 0.51 | 1.03 |
| Former smokers | -0.23 | 0.39 | 0.556 | 0.79 | 0.37 | 1.71 |
| **Alcohol consumption** |  |  |  |  |  |  |
| Non-drinkers | Reference |  |  |  |  |  |
| Current drinkers | 0.36 | 0.14 | 0.010 | 1.43 | 1.09 | 1.89 |
| Former drinkers | -1.58 | 1.01 | 0.117 | 0.21 | 0.03 | 1.49 |
| **Family history of hypertension** |  |  |  |  |  |  |
| With family history of hypertension | Reference |  |  |  |  |  |
| Without family history of hypertension | 0.24 | 0.11 | 0.035 | 1.27 | 1.02 | 1.58 |

*Note*: SBP, systolic blood pressure; DBP, diastolic blood pressure; BMI, body mass index; IRR, incidence rate ratios; CI, confidence interval; SE=standard error.

# 7. Supplemental Table

## **Table S7. Incidence rate ratios of hypertension by the total score of family-related life events**

| **Variable** | **Estimate** | **SE** | **P** | **IRR** | **95% CI** | |
| --- | --- | --- | --- | --- | --- | --- |
|  |  |  |  |  | **Lower** | **Upper** |
| Crude Poisson model |  |  |  |  |  |  |
| Intercept | -3.09 | 0.06 | <0.001 | 0.05 | 0.04 | 0.05 |
| Total score of family-related life events | -0.02 | 0.02 | 0.189 | 0.98 | 0.95 | 1.01 |
| Age- and sex-adjusted Poisson model |  |  |  |  |  |  |
| Intercept | -4.38 | 0.27 | <0.001 | 0.01 | 0.01 | 0.02 |
| Age | 0.04 | 0.01 | <0.001 | 1.04 | 1.03 | 1.05 |
| Sex |  |  |  |  |  |  |
| Male | Reference |  |  |  |  |  |
| Female | -0.63 | 0.11 | <0.001 | 0.53 | 0.43 | 0.66 |
| Total score of family-related life events | 0.00 | 0.02 | 0.934 | 1.00 | 0.97 | 1.03 |
| Multivariable Poisson model |  |  |  |  |  |  |
| Intercept | -15.83 | 0.94 | <0.001 | 0.00 | 0.00 | 0.00 |
| Age | 0.03 | 0.01 | <0.001 | 1.03 | 1.01 | 1.05 |
| **Sex** |  |  |  |  |  |  |
| Male | Reference |  |  |  |  |  |
| Female | -0.02 | 0.14 | 0.869 | 0.98 | 0.74 | 1.30 |
| BMI | 0.01 | 0.01 | 0.436 | 1.01 | 0.99 | 1.02 |
| SBP | 0.08 | 0.01 | <0.001 | 1.08 | 1.06 | 1.10 |
| DBP | 0.04 | 0.01 | <0.001 | 1.04 | 1.02 | 1.06 |
| Total score of family-related life events | 0.00 | 0.02 | 0.889 | 1.00 | 0.97 | 1.03 |
| **Marital status** |  |  |  |  |  |  |
| Single | Reference |  |  |  |  |  |
| Married | -0.08 | 0.19 | 0.667 | 0.92 | 0.64 | 1.34 |
| Widowed or divorced | 0.28 | 0.35 | 0.423 | 1.33 | 0.66 | 2.65 |
| **Educational level** |  |  |  |  |  |  |
| High school or below | Reference |  |  |  |  |  |
| Bachelor | -0.45 | 0.22 | 0.045 | 0.64 | 0.41 | 0.99 |
| Postgraduate or above | -0.76 | 0.26 | 0.003 | 0.47 | 0.28 | 0.77 |
| **Occupation** |  |  |  |  |  |  |
| Government officials | Reference |  |  |  |  |  |
| Other governmental employees | -0.36 | 0.29 | 0.212 | 0.70 | 0.40 | 1.23 |
| Teachers or researchers | -0.17 | 0.25 | 0.498 | 0.84 | 0.51 | 1.39 |
| Police | -0.19 | 0.30 | 0.520 | 0.82 | 0.46 | 1.49 |
| Medical staff | -0.25 | 0.24 | 0.294 | 0.78 | 0.49 | 1.24 |
| **Position title** |  |  |  |  |  |  |
| Junior professional staff | Reference |  |  |  |  |  |
| Intermediate professional staff | -0.02 | 0.14 | 0.906 | 0.98 | 0.74 | 1.30 |
| Senior professional staff | -0.24 | 0.20 | 0.233 | 0.79 | 0.53 | 1.16 |
| **Physical activity** |  |  |  |  |  |  |
| <1/week | Reference |  |  |  |  |  |
| 1-2/week | -0.33 | 0.14 | 0.017 | 0.72 | 0.55 | 0.94 |
| >3/week | -0.49 | 0.14 | <0.001 | 0.61 | 0.47 | 0.80 |
| **Smoking** |  |  |  |  |  |  |
| Nonsmokers | Reference |  |  |  |  |  |
| Current Smokers | -0.30 | 0.18 | 0.095 | 0.74 | 0.53 | 1.05 |
| Former smokers | -0.18 | 0.39 | 0.645 | 0.84 | 0.39 | 1.80 |
| **Alcohol consumption** |  |  |  |  |  |  |
| Non-drinkers | Reference |  |  |  |  |  |
| Current drinkers | 0.37 | 0.14 | 0.009 | 1.44 | 1.10 | 1.90 |
| Former drinkers | -1.55 | 1.01 | 0.124 | 0.21 | 0.03 | 1.53 |
| **Family history of hypertension** |  |  |  |  |  |  |
| With family history of hypertension | Reference |  |  |  |  |  |
| Without family history of hypertension | 0.24 | 0.11 | 0.030 | 1.28 | 1.02 | 1.59 |

*Note*: SBP, systolic blood pressure; DBP, diastolic blood pressure; BMI, body mass index; IRR, incidence rate ratios; CI, confidence interval; SE=standard error.

# 8. Supplemental Table

## **Table S8. Incidence rate ratios of hypertension by total score of other personal life events**

| **Variable** | **Estimate** | **SE** | **P** | **IRR** | **95% CI** | |
| --- | --- | --- | --- | --- | --- | --- |
|  |  |  |  |  | **Lower** | **Upper** |
| Crude Poisson model |  |  |  |  |  |  |
| Intercept | -3.18 | 0.06 | <0.001 | 0.04 | 0.04 | 0.05 |
| Total score of other personal life events | 0.03 | 0.02 | 0.170 | 1.03 | 0.99 | 1.08 |
| Age- and sex-adjusted Poisson model |  |  |  |  |  |  |
| Intercept | -4.62 | 0.27 | <0.001 | 0.01 | 0.01 | 0.02 |
| Age | 0.04 | 0.01 | <0.001 | 1.05 | 1.03 | 1.06 |
| **Sex** |  |  |  |  |  |  |
| Male | Reference |  |  |  |  |  |
| Female | -0.62 | 0.11 | <0.001 | 0.54 | 0.44 | 0.67 |
| Total score of other personal life events | 0.07 | 0.02 | 0.001 | 1.07 | 1.03 | 1.12 |
| Multivariable Poisson model |  |  |  |  |  |  |
| Intercept | -16.13 | 0.94 | <0.001 | 0.00 | 0.00 | 0.00 |
| Age | 0.03 | 0.01 | <0.001 | 1.03 | 1.02 | 1.05 |
| **Sex** |  |  |  |  |  |  |
| Male | Reference |  |  |  |  |  |
| Female | -0.01 | 0.14 | 0.961 | 0.99 | 0.75 | 1.32 |
| BMI | 0.01 | 0.01 | 0.431 | 1.01 | 0.99 | 1.02 |
| SBP | 0.08 | 0.01 | <0.001 | 1.08 | 1.06 | 1.10 |
| DBP | 0.04 | 0.01 | <0.001 | 1.04 | 1.02 | 1.06 |
| Total score of other personal life events | 0.07 | 0.02 | 0.001 | 1.08 | 1.03 | 1.12 |
| **Marital status** |  |  |  |  |  |  |
| Single | Reference |  |  |  |  |  |
| Married | -0.05 | 0.18 | 0.790 | 0.95 | 0.66 | 1.36 |
| Widowed or divorced | 0.23 | 0.35 | 0.498 | 1.26 | 0.64 | 2.49 |
| **Educational level** |  |  |  |  |  |  |
| High school or below | Reference |  |  |  |  |  |
| Bachelor | -0.45 | 0.22 | 0.043 | 0.64 | 0.41 | 0.99 |
| Postgraduate or above | -0.76 | 0.26 | 0.003 | 0.47 | 0.28 | 0.77 |
| **Occupation** |  |  |  |  |  |  |
| Government officials | Reference |  |  |  |  |  |
| Other governmental employees | -0.34 | 0.29 | 0.234 | 0.71 | 0.41 | 1.25 |
| Teachers or researchers | -0.18 | 0.25 | 0.467 | 0.83 | 0.50 | 1.37 |
| Police | -0.22 | 0.30 | 0.466 | 0.80 | 0.45 | 1.45 |
| Medical staff | -0.25 | 0.24 | 0.294 | 0.78 | 0.49 | 1.24 |
| **Position title** |  |  |  |  |  |  |
| Junior professional staff | Reference |  |  |  |  |  |
| Intermediate professional staff | -0.00 | 0.14 | 0.989 | 1.00 | 0.76 | 1.32 |
| Senior professional staff | -0.23 | 0.20 | 0.237 | 0.79 | 0.54 | 1.17 |
| **Physical activity** |  |  |  |  |  |  |
| <1/week | Reference |  |  |  |  |  |
| 1-2/week | -0.32 | 0.14 | 0.021 | 0.72 | 0.55 | 0.95 |
| >3/week | -0.48 | 0.14 | <0.001 | 0.62 | 0.47 | 0.80 |
| **Smoking** |  |  |  |  |  |  |
| Nonsmokers | Reference |  |  |  |  |  |
| Current Smokers | -0.30 | 0.18 | 0.090 | 0.74 | 0.52 | 1.05 |
| Former smokers | -0.19 | 0.39 | 0.619 | 0.82 | 0.38 | 1.77 |
| **Alcohol consumption** |  |  |  |  |  |  |
| Non-drinkers | Reference |  |  |  |  |  |
| Current drinkers | 0.36 | 0.14 | 0.011 | 1.43 | 1.09 | 1.88 |
| Former drinkers | -1.56 | 1.01 | 0.121 | 0.21 | 0.03 | 1.51 |
| **Family history of hypertension** |  |  |  |  |  |  |
| With family history of hypertension | Reference |  |  |  |  |  |
| Without family history of hypertension | 0.24 | 0.11 | 0.034 | 1.27 | 1.02 | 1.58 |

*Note*: SBP, systolic blood pressure; DBP, diastolic blood pressure; BMI, body mass index; IRR, incidence rate ratios; CI, confidence interval; SE=standard error.

# 9. Supplemental Table

## **Table S9. Incidence rate ratios of hypertension by positive and negative work-related life events**

| **Variable** | **Estimate** | **SE** | **P** | **IRR** | **95% CI** | |
| --- | --- | --- | --- | --- | --- | --- |
|  |  |  |  |  | **Lower** | **Upper** |
| Crude Poisson model |  |  |  |  |  |  |
| Intercept | -3.17 | 0.06 | <0.001 | 0.04 | 0.04 | 0.05 |
| Score of positive work-related life events | -0.18 | 0.08 | 0.022 | 0.84 | 0.72 | 0.97 |
| Score of negative work-related life events | 0.07 | 0.02 | 0.003 | 1.07 | 1.02 | 1.12 |
| Age- and sex-adjusted Poisson model |  |  |  |  |  |  |
| Intercept | -4.53 | 0.27 | <0.001 | 0.01 | 0.01 | 0.02 |
| Age | 0.04 | 0.01 | <0.001 | 1.04 | 1.03 | 1.06 |
| **Sex** |  |  |  |  |  |  |
| Male | Reference |  |  |  |  |  |
| Female | -0.64 | 0.11 | <0.001 | 0.53 | 0.42 | 0.65 |
| Score of positive work-related life events | -0.14 | 0.08 | 0.077 | 0.87 | 0.75 | 1.01 |
| Score of negative work-related life events | 0.10 | 0.02 | <0.001 | 1.11 | 1.06 | 1.16 |
| Multivariable Poisson model |  |  |  |  |  |  |
| Intercept | -16.25 | 0.95 | <0.001 | 0.00 | 0.00 | 0.00 |
| Age | 0.03 | 0.01 | <0.001 | 1.04 | 1.02 | 1.05 |
| **Sex** |  |  |  |  |  |  |
| Male | Reference |  |  |  |  |  |
| Female | -0.04 | 0.14 | 0.771 | 0.96 | 0.72 | 1.27 |
| BMI | 0.01 | 0.01 | 0.454 | 1.01 | 0.99 | 1.02 |
| SBP | 0.08 | 0.01 | <0.001 | 1.08 | 1.07 | 1.10 |
| DBP | 0.04 | 0.01 | <0.001 | 1.04 | 1.02 | 1.06 |
| Score of positive work-related life events | -0.13 | 0.08 | 0.085 | 0.88 | 0.75 | 1.02 |
| Score of negative work-related life events | 0.12 | 0.02 | <0.001 | 1.13 | 1.07 | 1.18 |
| **Marital status** |  |  |  |  |  |  |
| Single | Reference |  |  |  |  |  |
| Married | -0.10 | 0.18 | 0.602 | 0.91 | 0.64 | 1.30 |
| Widowed or divorced | 0.18 | 0.35 | 0.609 | 1.19 | 0.60 | 2.36 |
| **Educational level** |  |  |  |  |  |  |
| High school or below | Reference |  |  |  |  |  |
| Bachelor | -0.46 | 0.22 | 0.041 | 0.63 | 0.41 | 0.98 |
| Postgraduate or above | -0.77 | 0.26 | 0.003 | 0.46 | 0.28 | 0.77 |
| **Occupation** |  |  |  |  |  |  |
| Government officials | Reference |  |  |  |  |  |
| Other governmental employees | -0.28 | 0.29 | 0.333 | 0.76 | 0.43 | 1.33 |
| Teachers or researchers | -0.15 | 0.25 | 0.567 | 0.86 | 0.52 | 1.42 |
| Police | -0.20 | 0.30 | 0.516 | 0.82 | 0.46 | 1.48 |
| Medical staff | -0.19 | 0.24 | 0.428 | 0.83 | 0.52 | 1.32 |
| **Position title** |  |  |  |  |  |  |
| Junior professional staff | Reference |  |  |  |  |  |
| Intermediate professional staff | -0.02 | 0.14 | 0.884 | 0.98 | 0.74 | 1.29 |
| Senior professional staff | -0.24 | 0.20 | 0.229 | 0.79 | 0.53 | 1.16 |
| **Physical activity** |  |  |  |  |  |  |
| <1/week | Reference |  |  |  |  |  |
| 1-2/week | -0.30 | 0.14 | 0.034 | 0.74 | 0.56 | 0.98 |
| >3/week | -0.45 | 0.14 | 0.001 | 0.64 | 0.49 | 0.83 |
| **Smoking** |  |  |  |  |  |  |
| Nonsmokers | Reference |  |  |  |  |  |
| Current Smokers | -0.35 | 0.18 | 0.046 | 0.70 | 0.50 | 0.99 |
| Former smokers | -0.26 | 0.39 | 0.509 | 0.77 | 0.36 | 1.67 |
| **Alcohol consumption** |  |  |  |  |  |  |
| Non-drinkers | Reference |  |  |  |  |  |
| Current drinkers | 0.37 | 0.14 | 0.008 | 1.45 | 1.10 | 1.91 |
| Former drinkers | -1.63 | 1.01 | 0.107 | 0.20 | 0.03 | 1.42 |
| **Family history of hypertension** |  |  |  |  |  |  |
| With family history of hypertension | Reference |  |  |  |  |  |
| Without family history of hypertension | 0.23 | 0.11 | 0.044 | 1.25 | 1.01 | 1.56 |

*Note*: SBP, systolic blood pressure; DBP, diastolic blood pressure; BMI, body mass index; IRR, incidence rate ratios; CI, confidence interval; SE=standard error.

# 10. Supplemental Table

## **Table S10. Incidence rate ratios of hypertension by positive and negative family-related life events**

| **Variable** | **Estimate** | **SE** | **P** | **IRR** | **95% CI** | |
| --- | --- | --- | --- | --- | --- | --- |
|  |  |  |  |  | **Lower** | **Upper** |
| Crude Poisson model |  |  |  |  |  |  |
| Intercept | -3.08 | 0.06 | <0.001 | 0.05 | 0.04 | 0.05 |
| Score of positive family-related life events | -0.21 | 0.05 | <0.001 | 0.81 | 0.74 | 0.89 |
| Score of negative family-related life events | 0.05 | 0.02 | 0.009 | 1.05 | 1.01 | 1.08 |
| Age- and sex-adjusted Poisson model |  |  |  |  |  |  |
| Intercept | -4.21 | 0.27 | <0.001 | 0.01 | 0.01 | 0.03 |
| Age | 0.04 | 0.01 | <0.001 | 1.04 | 1.03 | 1.05 |
| Sex |  |  |  |  |  |  |
| Male | Reference |  |  |  |  |  |
| Female | -0.63 | 0.11 | <0.001 | 0.53 | 0.43 | 0.66 |
| Score of positive family-related life events | -0.15 | 0.05 | 0.001 | 0.86 | 0.78 | 0.94 |
| Score of negative family-related life events | 0.05 | 0.02 | 0.005 | 1.05 | 1.01 | 1.09 |
| Multivariable Poisson model |  |  |  |  |  |  |
| Intercept | -15.73 | 0.94 | <0.001 | 0.00 | 0.00 | 0.00 |
| Age | 0.03 | 0.01 | 0.002 | 1.03 | 1.01 | 1.04 |
| Sex |  |  |  |  |  |  |
| Male | Reference |  |  |  |  |  |
| Female | -0.02 | 0.14 | 0.916 | 0.98 | 0.74 | 1.31 |
| BMI | 0.01 | 0.01 | 0.409 | 1.01 | 0.99 | 1.02 |
| SBP | 0.08 | 0.01 | <0.001 | 1.08 | 1.07 | 1.10 |
| DBP | 0.04 | 0.01 | <0.001 | 1.04 | 1.02 | 1.06 |
| Score of positive family-related life events | -0.13 | 0.05 | 0.004 | 0.87 | 0.80 | 0.96 |
| Score of negative family-related life events | 0.04 | 0.02 | 0.026 | 1.04 | 1.00 | 1.08 |
| **Marital status** |  |  |  |  |  |  |
| Single | Reference |  |  |  |  |  |
| Married | 0.03 | 0.19 | 0.895 | 1.03 | 0.70 | 1.49 |
| Widowed or divorced | 0.25 | 0.35 | 0.472 | 1.29 | 0.64 | 2.59 |
| **Educational level** |  |  |  |  |  |  |
| High school or below | Reference |  |  |  |  |  |
| Bachelor | -0.45 | 0.22 | 0.043 | 0.64 | 0.41 | 0.99 |
| Postgraduate or above | -0.73 | 0.26 | 0.004 | 0.48 | 0.29 | 0.79 |
| **Occupation** |  |  |  |  |  |  |
| Government officials | Reference |  |  |  |  |  |
| Other governmental employees | -0.36 | 0.29 | 0.207 | 0.70 | 0.40 | 1.22 |
| Teachers or researchers | -0.16 | 0.25 | 0.518 | 0.85 | 0.52 | 1.40 |
| Police | -0.22 | 0.30 | 0.475 | 0.81 | 0.45 | 1.46 |
| Medical staff | -0.24 | 0.24 | 0.326 | 0.79 | 0.49 | 1.26 |
| **Position title** |  |  |  |  |  |  |
| Junior professional staff | Reference |  |  |  |  |  |
| Intermediate professional staff | -0.03 | 0.14 | 0.859 | 0.98 | 0.74 | 1.29 |
| Senior professional staff | -0.26 | 0.20 | 0.190 | 0.77 | 0.52 | 1.14 |
| **Physical activity** |  |  |  |  |  |  |
| <1/week | Reference |  |  |  |  |  |
| 1-2/week | -0.35 | 0.14 | 0.013 | 0.71 | 0.54 | 0.93 |
| >3/week | -0.48 | 0.13 | <0.001 | 0.62 | 0.47 | 0.80 |
| **Smoking** |  |  |  |  |  |  |
| Nonsmokers | Reference |  |  |  |  |  |
| Current Smokers | -0.30 | 0.18 | 0.091 | 0.74 | 0.52 | 1.05 |
| Former smokers | -0.18 | 0.39 | 0.639 | 0.83 | 0.39 | 1.79 |
| **Alcohol consumption** |  |  |  |  |  |  |
| Non-drinkers | Reference |  |  |  |  |  |
| Current drinkers | 0.37 | 0.14 | 0.009 | 1.44 | 1.10 | 1.90 |
| Former drinkers | -1.53 | 1.01 | 0.128 | 0.22 | 0.03 | 1.56 |
| **Family history of hypertension** |  |  |  |  |  |  |
| With family history of hypertension | Reference |  |  |  |  |  |
| Without family history of hypertension | 0.24 | 0.11 | 0.036 | 1.27 | 1.02 | 1.58 |

*Note*: SBP, systolic blood pressure; DBP, diastolic blood pressure; BMI, body mass index; IRR, incidence rate ratios; CI, confidence interval; SE=standard error.

# 11. Supplemental Table

## **Table S11. Incidence rate ratios of hypertension by other positive and negative personal life events**

| **Variable** | **Estimate** | **SE** | ***P*** | **IRR** | **95% CI** | |
| --- | --- | --- | --- | --- | --- | --- |
|  |  |  |  |  | **Lower** | **Upper** |
| Crude Poisson model |  |  |  |  |  |  |
| Intercept | -3.16 | 0.06 | <0.001 | 0.04 | 0.04 | 0.05 |
| Score of other positive personal life events | -0.14 | 0.07 | 0.056 | 0.87 | 0.76 | 1.00 |
| Score of other negative personal life events | 0.07 | 0.02 | 0.004 | 1.07 | 1.02 | 1.13 |
| Age- and sex-adjusted Poisson model |  |  |  |  |  |  |
| Intercept | -4.54 | 0.28 | <0.001 | 0.01 | 0.01 | 0.02 |
| Age | 0.04 | 0.01 | <0.001 | 1.04 | 1.03 | 1.06 |
| **Sex** |  |  |  |  |  |  |
| Male | Reference |  |  |  |  |  |
| Female | -0.62 | 0.11 | <0.001 | 0.54 | 0.43 | 0.66 |
| Score of other positive personal life events | -0.01 | 0.07 | 0.898 | 0.99 | 0.86 | 1.14 |
| Score of other negative personal life events | 0.08 | 0.02 | <0.001 | 1.09 | 1.04 | 1.14 |
| Multivariable Poisson model |  |  |  |  |  |  |
| Intercept | -16.05 | 0.94 | <0.001 | 0.00 | 0.00 | 0.00 |
| Age | 0.03 | 0.01 | <0.001 | 1.03 | 1.02 | 1.05 |
| **Sex** |  |  |  |  |  |  |
| Male | Reference |  |  |  |  |  |
| Female | -0.01 | 0.14 | 0.929 | 0.99 | 0.74 | 1.31 |
| BMI | 0.01 | 0.01 | 0.451 | 1.01 | 0.99 | 1.02 |
| SBP | 0.08 | 0.01 | <0.001 | 1.08 | 1.06 | 1.10 |
| DBP | 0.04 | 0.01 | <0.001 | 1.04 | 1.02 | 1.06 |
| Score of other positive personal life events | -0.03 | 0.07 | 0.676 | 0.97 | 0.84 | 1.12 |
| Score of other negative personal life events | 0.09 | 0.02 | <0.001 | 1.10 | 1.05 | 1.15 |
| **Marital status** |  |  |  |  |  |  |
| Single | Reference |  |  |  |  |  |
| Married | -0.08 | 0.18 | 0.653 | 0.92 | 0.64 | 1.32 |
| Widowed or divorced | 0.20 | 0.35 | 0.560 | 1.22 | 0.62 | 2.41 |
| **Educational level** |  |  |  |  |  |  |
| High school or below | Reference |  |  |  |  |  |
| Bachelor | -0.45 | 0.22 | 0.045 | 0.64 | 0.41 | 0.99 |
| Postgraduate or above | -0.75 | 0.26 | 0.004 | 0.47 | 0.29 | 0.78 |
| **Occupation** |  |  |  |  |  |  |
| Government officials | Reference |  |  |  |  |  |
| Other governmental employees | -0.34 | 0.29 | 0.237 | 0.71 | 0.41 | 1.25 |
| Teachers or researchers | -0.18 | 0.25 | 0.482 | 0.84 | 0.51 | 1.38 |
| Police | -0.23 | 0.30 | 0.451 | 0.80 | 0.44 | 1.44 |
| Medical staff | -0.25 | 0.24 | 0.294 | 0.78 | 0.49 | 1.24 |
| **Position title** |  |  |  |  |  |  |
| Junior professional staff | Reference |  |  |  |  |  |
| Intermediate professional staff | -0.02 | 0.14 | 0.907 | 0.98 | 0.75 | 1.30 |
| Senior professional staff | -0.25 | 0.20 | 0.201 | 0.78 | 0.53 | 1.14 |
| **Physical activity** |  |  |  |  |  |  |
| <1/week | Reference |  |  |  |  |  |
| 1-2/week | -0.32 | 0.14 | 0.024 | 0.73 | 0.55 | 0.96 |
| >3/week | -0.48 | 0.14 | <0.001 | 0.62 | 0.47 | 0.80 |
| **Smoking** |  |  |  |  |  |  |
| Nonsmokers | Reference |  |  |  |  |  |
| Current Smokers | -0.30 | 0.18 | 0.088 | 0.74 | 0.52 | 1.05 |
| Former smokers | -0.21 | 0.39 | 0.598 | 0.81 | 0.38 | 1.75 |
| **Alcohol consumption** |  |  |  |  |  |  |
| Non-drinkers | Reference |  |  |  |  |  |
| Current drinkers | 0.36 | 0.14 | 0.010 | 1.44 | 1.09 | 1.89 |
| Former drinkers | -1.57 | 1.01 | 0.119 | 0.21 | 0.03 | 1.50 |
| **Family history of hypertension** |  |  |  |  |  |  |
| With family history of hypertension | Reference |  |  |  |  |  |
| Without family history of hypertension | 0.24 | 0.11 | 0.033 | 1.27 | 1.02 | 1.58 |

*Note*: SBP, systolic blood pressure; DBP, diastolic blood pressure; BMI, body mass index; IRR, incidence rate ratios; CI, confidence interval; SE=standard error.

# 12. Supplemental Table

## **Table S12. Incidence rate ratios of hypertension by quartiles of all life events**

| **Variable** | **Estimate** | **SE** | **P** | **IRR** | **95% CI** | |
| --- | --- | --- | --- | --- | --- | --- |
|  |  |  |  |  | **Lower** | **Upper** |
| Intercept | -16.29 | 0.95 | <0.001 | 0.00 | 0.00 | 0.00 |
| Age | 0.03 | 0.01 | 0.001 | 1.03 | 1.01 | 1.05 |
| **Sex** |  |  |  |  |  |  |
| Male | Reference |  |  |  |  |  |
| Female | -0.04 | 0.14 | 0.758 | 0.96 | 0.72 | 1.27 |
| BMI | 0.01 | 0.01 | 0.433 | 1.01 | 0.99 | 1.02 |
| SBP | 0.08 | 0.01 | <0.001 | 1.08 | 1.07 | 1.10 |
| DBP | 0.04 | 0.01 | <0.001 | 1.04 | 1.02 | 1.06 |
| **Total score of negative life events** |  |  |  |  |  |  |
| Quartile 1 | Reference |  |  |  |  |  |
| Quartile 2 | 0.79 | 0.15 | <0.001 | 2.19 | 1.63 | 2.94 |
| Quartile 3 | 0.92 | 0.16 | <0.001 | 2.51 | 1.83 | 3.44 |
| Quartile 4 | 1.13 | 0.16 | <0.001 | 3.09 | 2.26 | 4.23 |
| **Total score of positive life events** |  |  |  |  |  |  |
| Quartile 1 | Reference |  |  |  |  |  |
| Quartile 2 | -0.61 | 0.15 | <0.001 | 0.54 | 0.41 | 0.73 |
| Quartile 3 | -0.68 | 0.17 | <0.001 | 0.51 | 0.36 | 0.71 |
| **Marital status** |  |  |  |  |  |  |
| Single | Reference |  |  |  |  |  |
| Married | -0.11 | 0.18 | 0.559 | 0.90 | 0.63 | 1.29 |
| Widowed or divorced | 0.09 | 0.35 | 0.797 | 1.09 | 0.55 | 2.16 |
| **Educational level** |  |  |  |  |  |  |
| High school or below | Reference |  |  |  |  |  |
| Bachelor | -0.52 | 0.22 | 0.020 | 0.59 | 0.38 | 0.92 |
| Postgraduate or above | -0.79 | 0.26 | 0.002 | 0.45 | 0.27 | 0.75 |
| **Occupation** |  |  |  |  |  |  |
| Government officials | Reference |  |  |  |  |  |
| Other governmental employees | -0.23 | 0.29 | 0.428 | 0.80 | 0.45 | 1.40 |
| Teachers or researchers | -0.13 | 0.25 | 0.610 | 0.88 | 0.53 | 1.45 |
| Police | -0.11 | 0.30 | 0.707 | 0.89 | 0.49 | 1.61 |
| Medical staff | -0.14 | 0.24 | 0.562 | 0.87 | 0.54 | 1.39 |
| **Position title** |  |  |  |  |  |  |
| Junior professional staff | Reference |  |  |  |  |  |
| Intermediate professional staff | -0.04 | 0.14 | 0.773 | 0.96 | 0.73 | 1.27 |
| Senior professional staff | -0.23 | 0.20 | 0.256 | 0.80 | 0.54 | 1.18 |
| **Physical activity** |  |  |  |  |  |  |
| <1/week | Reference |  |  |  |  |  |
| 1-2/week | -0.28 | 0.14 | 0.043 | 0.75 | 0.57 | 0.99 |
| >3/week | -0.41 | 0.14 | 0.003 | 0.67 | 0.51 | 0.87 |
| **Smoking** |  |  |  |  |  |  |
| Nonsmokers | Reference |  |  |  |  |  |
| Current Smokers | -0.34 | 0.18 | 0.054 | 0.71 | 0.50 | 1.01 |
| Former smokers | -0.25 | 0.39 | 0.529 | 0.78 | 0.36 | 1.68 |
| **Alcohol consumption** |  |  |  |  |  |  |
| Non-drinkers | Reference |  |  |  |  |  |
| Current drinkers | 0.35 | 0.14 | 0.012 | 1.42 | 1.08 | 1.87 |
| Former drinkers | -1.52 | 1.01 | 0.131 | 0.22 | 0.03 | 1.57 |
| **Family history of hypertension** |  |  |  |  |  |  |
| No | Reference |  |  |  |  |  |
| Yes | 0.20 | 0.11 | 0.081 | 1.22 | 0.98 | 1.52 |

*Note*: SBP, systolic blood pressure; DBP, diastolic blood pressure; BMI, body mass index; IRR, incidence rate ratios; CI, confidence interval; SE=standard error.

# 13. Supplemental Table

## **Table S13. Incidence rate ratios of hypertension by quartiles of positive and negative life events**

| **Variable** | **Estimate** | **SE** | **P** | **IRR** | **95% CI** | |
| --- | --- | --- | --- | --- | --- | --- |
|  |  |  |  |  | **Lower** | **Upper** |
| Intercept | -16.29 | 0.95 | <0.001 | 0.00 | 0.00 | 0.00 |
| Age | 0.03 | 0.01 | 0.001 | 1.03 | 1.01 | 1.05 |
| **Sex** |  |  |  |  |  |  |
| Male | Reference |  |  |  |  |  |
| Female | -0.04 | 0.14 | 0.758 | 0.96 | 0.72 | 1.27 |
| BMI | 0.01 | 0.01 | 0.433 | 1.01 | 0.99 | 1.02 |
| SBP | 0.08 | 0.01 | <0.001 | 1.08 | 1.07 | 1.10 |
| DBP | 0.04 | 0.01 | <0.001 | 1.04 | 1.02 | 1.06 |
| **Total score of negative life events** |  |  |  |  |  |  |
| Quartile 1 | Reference |  |  |  |  |  |
| Quartile 2 | 0.79 | 0.15 | <0.001 | 2.19 | 1.63 | 2.94 |
| Quartile 3 | 0.92 | 0.16 | <0.001 | 2.51 | 1.83 | 3.44 |
| Quartile 4 | 1.13 | 0.16 | <0.001 | 3.09 | 2.26 | 4.23 |
| **Total score of positive life events** |  |  |  |  |  |  |
| Quartile 1 and 2 | Reference |  |  |  |  |  |
| Quartile 3 | -0.61 | 0.15 | <0.001 | 0.54 | 0.41 | 0.73 |
| Quartile 4 | -0.68 | 0.17 | <0.001 | 0.51 | 0.36 | 0.71 |
| **Marital status** |  |  |  |  |  |  |
| Single | Reference |  |  |  |  |  |
| Married | -0.11 | 0.18 | 0.559 | 0.90 | 0.63 | 1.29 |
| Widowed or divorced | 0.09 | 0.35 | 0.797 | 1.09 | 0.55 | 2.16 |
| **Educational level** |  |  |  |  |  |  |
| High school or below | Reference |  |  |  |  |  |
| Bachelor | -0.52 | 0.22 | 0.020 | 0.59 | 0.38 | 0.92 |
| Postgraduate or above | -0.79 | 0.26 | 0.002 | 0.45 | 0.27 | 0.75 |
| **Occupation** |  |  |  |  |  |  |
| Government officials | Reference |  |  |  |  |  |
| Other governmental employees | -0.23 | 0.29 | 0.428 | 0.80 | 0.45 | 1.40 |
| Teachers or researchers | -0.13 | 0.25 | 0.610 | 0.88 | 0.53 | 1.45 |
| Police | -0.11 | 0.30 | 0.707 | 0.89 | 0.49 | 1.61 |
| Medical staff | -0.14 | 0.24 | 0.562 | 0.87 | 0.54 | 1.39 |
| **Position title** |  |  |  |  |  |  |
| Junior professional staff | Reference |  |  |  |  |  |
| Intermediate professional staff | -0.04 | 0.14 | 0.773 | 0.96 | 0.73 | 1.27 |
| Senior professional staff | -0.23 | 0.20 | 0.256 | 0.80 | 0.54 | 1.18 |
| **Physical activity** |  |  |  |  |  |  |
| <1/week | Reference |  |  |  |  |  |
| 1-2/week | -0.28 | 0.14 | 0.043 | 0.75 | 0.57 | 0.99 |
| >3/week | -0.41 | 0.14 | 0.003 | 0.67 | 0.51 | 0.87 |
| **Smoking** |  |  |  |  |  |  |
| Nonsmokers | Reference |  |  |  |  |  |
| Current Smokers | -0.34 | 0.18 | 0.054 | 0.71 | 0.50 | 1.01 |
| Former smokers | -0.25 | 0.39 | 0.529 | 0.78 | 0.36 | 1.68 |
| **Alcohol consumption** |  |  |  |  |  |  |
| Non-drinkers | Reference |  |  |  |  |  |
| Current drinkers | 0.35 | 0.14 | 0.012 | 1.42 | 1.08 | 1.87 |
| Former drinkers | -1.52 | 1.01 | 0.131 | 0.22 | 0.03 | 1.57 |
| **Family history of hypertension** |  |  |  |  |  |  |
| No | Reference |  |  |  |  |  |
| Yes | 0.20 | 0.11 | 0.081 | 1.22 | 0.98 | 1.52 |

*Note*: SBP, systolic blood pressure; DBP, diastolic blood pressure; BMI, body mass index; IRR, incidence rate ratios; CI, confidence interval; SE=standard error.

# 14. Supplemental Table

## **Table S14. Incidence rate ratios of hypertension by quartiles of work-related life events**

| **Variable** | **Estimate** | **SE** | **P** | **IRR** | **95% CI** | |
| --- | --- | --- | --- | --- | --- | --- |
|  |  |  |  |  | **Lower** | **Upper** |
| Intercept | -16.37 | 0.95 | <0.001 | 0.00 | 0.00 | 0.00 |
| Age | 0.04 | 0.01 | <0.001 | 1.04 | 1.02 | 1.05 |
| **Sex** |  |  |  |  |  |  |
| Male | Reference |  |  |  |  |  |
| Female | -0.02 | 0.14 | 0.909 | 0.98 | 0.74 | 1.30 |
| BMI | 0.01 | 0.01 | 0.409 | 1.01 | 0.99 | 1.02 |
| SBP | 0.08 | 0.01 | <0.001 | 1.08 | 1.07 | 1.10 |
| DBP | 0.04 | 0.01 | <0.001 | 1.04 | 1.02 | 1.06 |
| **Total score of life events** |  |  |  |  |  |  |
| Quartile 1 | Reference |  |  |  |  |  |
| Quartile 2 | 0.35 | 0.13 | 0.009 | 1.41 | 1.09 | 1.83 |
| Quartile 3 | 0.50 | 0.14 | <0.001 | 1.65 | 1.25 | 2.18 |
| **Marital status** |  |  |  |  |  |  |
| Single | Reference |  |  |  |  |  |
| Married | -0.04 | 0.18 | 0.809 | 0.96 | 0.67 | 1.37 |
| Widowed or divorced | 0.28 | 0.35 | 0.422 | 1.32 | 0.67 | 2.60 |
| **Educational level** |  |  |  |  |  |  |
| High school or below | Reference |  |  |  |  |  |
| Bachelor | -0.48 | 0.22 | 0.033 | 0.62 | 0.40 | 0.96 |
| Postgraduate or above | -0.82 | 0.26 | 0.001 | 0.44 | 0.27 | 0.73 |
| **Occupation** |  |  |  |  |  |  |
| Civil servant | Reference |  |  |  |  |  |
| Staff of state-owned enterprises | -0.27 | 0.29 | 0.356 | 0.77 | 0.44 | 1.35 |
| Teachers or researchers | -0.13 | 0.25 | 0.597 | 0.87 | 0.53 | 1.44 |
| Police | -0.17 | 0.30 | 0.565 | 0.84 | 0.47 | 1.52 |
| Medical staff | -0.17 | 0.24 | 0.484 | 0.85 | 0.53 | 1.35 |
| **Position title** |  |  |  |  |  |  |
| Junior professional staff | Reference |  |  |  |  |  |
| Intermediate professional staff | -0.03 | 0.14 | 0.853 | 0.97 | 0.74 | 1.29 |
| Senior professional staff | -0.28 | 0.20 | 0.163 | 0.76 | 0.51 | 1.12 |
| **Physical activity** |  |  |  |  |  |  |
| <1/week | Reference |  |  |  |  |  |
| 1-2/week | -0.33 | 0.14 | 0.019 | 0.72 | 0.55 | 0.95 |
| >3/week | -0.45 | 0.14 | 0.001 | 0.64 | 0.49 | 0.83 |
| **Smoking** |  |  |  |  |  |  |
| Nonsmokers | Reference |  |  |  |  |  |
| Current Smokers | -0.32 | 0.18 | 0.072 | 0.73 | 0.51 | 1.03 |
| Former smokers | -0.20 | 0.39 | 0.605 | 0.82 | 0.38 | 1.76 |
| **Alcohol consumption** |  |  |  |  |  |  |
| Non-drinkers | Reference |  |  |  |  |  |
| Current drinkers | 0.37 | 0.14 | 0.008 | 1.45 | 1.10 | 1.91 |
| Former drinkers | -1.61 | 1.01 | 0.110 | 0.20 | 0.03 | 1.44 |
| **Family history of hypertension** |  |  |  |  |  |  |
| No | Reference |  |  |  |  |  |
| Yes | 0.23 | 0.11 | 0.037 | 1.26 | 1.01 | 1.58 |

*Note*: SBP, systolic blood pressure; DBP, diastolic blood pressure; BMI, body mass index; IRR, incidence rate ratios; CI, confidence interval; SE=standard error.

# 15. Supplemental Table

## **Table S15. Incidence rate ratios of hypertension by quartiles of positive and negative work-related life events**

| **Variable** | **Estimate** | **SE** | **P** | **IRR** | **95% CI** | |
| --- | --- | --- | --- | --- | --- | --- |
|  |  |  |  |  | **Lower** | **Upper** |
| Intercept | -16.34 | 0.95 | <0.001 | 0.00 | 0.00 | 0.00 |
| Age | 0.03 | 0.01 | <0.001 | 1.04 | 1.02 | 1.05 |
| **Sex** |  |  |  |  |  |  |
| Male | Reference |  |  |  |  |  |
| Female | -0.03 | 0.14 | 0.852 | 0.97 | 0.73 | 1.29 |
| BMI | 0.01 | 0.01 | 0.467 | 1.01 | 0.99 | 1.02 |
| SBP | 0.08 | 0.01 | <0.001 | 1.08 | 1.07 | 1.10 |
| DBP | 0.04 | 0.01 | <0.001 | 1.04 | 1.02 | 1.06 |
| **Total score of negative work-related life events** |  |  |  |  |  |  |
| Quartile 1 and 2 | Reference |  |  |  |  |  |
| Quartile 3 | 0.56 | 0.13 | <0.001 | 1.74 | 1.35 | 2.24 |
| Quartile 4 | 0.70 | 0.15 | <0.001 | 2.01 | 1.48 | 2.71 |
| **Total score of positive work-related life events** |  |  |  |  |  |  |
| Quartile 1, 2 and 3 | Reference |  |  |  |  |  |
| Quartile 4 | -0.45 | 0.18 | 0.013 | 0.64 | 0.45 | 0.91 |
| **Marital status** |  |  |  |  |  |  |
| Single | Reference |  |  |  |  |  |
| Married | -0.09 | 0.18 | 0.627 | 0.92 | 0.64 | 1.31 |
| Widowed or divorced | 0.18 | 0.35 | 0.598 | 1.20 | 0.61 | 2.36 |
| **Educational level** |  |  |  |  |  |  |
| High school or below | Reference |  |  |  |  |  |
| Bachelor | -0.50 | 0.22 | 0.026 | 0.61 | 0.39 | 0.94 |
| Postgraduate or above | -0.82 | 0.26 | 0.001 | 0.44 | 0.27 | 0.73 |
| **Occupation** |  |  |  |  |  |  |
| Government officials | Reference |  |  |  |  |  |
| Other governmental employees | -0.24 | 0.29 | 0.400 | 0.78 | 0.45 | 1.38 |
| Teachers or researchers | -0.12 | 0.25 | 0.634 | 0.89 | 0.54 | 1.46 |
| Police | -0.16 | 0.30 | 0.592 | 0.85 | 0.47 | 1.54 |
| Medical staff | -0.16 | 0.24 | 0.515 | 0.86 | 0.53 | 1.37 |
| **Position title** |  |  |  |  |  |  |
| Junior professional staff | Reference |  |  |  |  |  |
| Intermediate professional staff | -0.02 | 0.14 | 0.870 | 0.98 | 0.74 | 1.29 |
| Senior professional staff | -0.23 | 0.20 | 0.247 | 0.79 | 0.54 | 1.17 |
| **Physical activity** |  |  |  |  |  |  |
| <1/week | Reference |  |  |  |  |  |
| 1-2/week | -0.30 | 0.14 | 0.033 | 0.74 | 0.56 | 0.98 |
| >3/week | -0.43 | 0.14 | 0.002 | 0.65 | 0.50 | 0.85 |
| **Smoking** |  |  |  |  |  |  |
| Nonsmokers | Reference |  |  |  |  |  |
| Current Smokers | -0.34 | 0.18 | 0.053 | 0.71 | 0.50 | 1.00 |
| Former smokers | -0.20 | 0.39 | 0.611 | 0.82 | 0.38 | 1.76 |
| **Alcohol consumption** |  |  |  |  |  |  |
| Non-drinkers | Reference |  |  |  |  |  |
| Current drinkers | 0.40 | 0.14 | 0.004 | 1.49 | 1.13 | 1.97 |
| Former drinkers | -1.69 | 1.01 | 0.095 | 0.19 | 0.03 | 1.34 |
| **Family history of hypertension** |  |  |  |  |  |  |
| No | Reference |  |  |  |  |  |
| Yes | 0.23 | 0.11 | 0.039 | 1.26 | 1.01 | 1.57 |

*Note*: SBP, systolic blood pressure; DBP, diastolic blood pressure; BMI, body mass index; IRR, incidence rate ratios; CI, confidence interval; SE=standard error.

# 16. Supplemental Table

## **Table S16. Incidence rate ratios of hypertension by quartiles of family-related life events**

| **Variable** | **Estimate** | **SE** | **P** | **IRR** | **95% CI** | |
| --- | --- | --- | --- | --- | --- | --- |
|  |  |  |  |  | **Lower** | **Lower** |
| Intercept | -15.89 | 0.94 | <0.001 | 0.00 | 0.00 | 0.00 |
| Age | 0.03 | 0.01 | <0.001 | 1.03 | 1.02 | 1.05 |
| **Sex** |  |  |  |  |  |  |
| Male | Reference |  |  |  |  |  |
| Female | -0.02 | 0.14 | 0.880 | 0.98 | 0.74 | 1.30 |
| BMI | 0.01 | 0.01 | 0.425 | 1.01 | 0.99 | 1.02 |
| SBP | 0.08 | 0.01 | <0.001 | 1.08 | 1.06 | 1.10 |
| DBP | 0.04 | 0.01 | <0.001 | 1.04 | 1.02 | 1.06 |
| **Total score of family-related life events** | |  |  |  |  |  |
| Quartile 1 | Reference |  |  |  |  |  |
| Quartile 2 | 0.18 | 0.17 | 0.270 | 1.20 | 0.87 | 1.66 |
| Quartile 3 | 0.11 | 0.15 | 0.488 | 1.11 | 0.82 | 1.50 |
| Quartile 4 | 0.09 | 0.15 | 0.538 | 1.10 | 0.82 | 1.47 |
| **Marital status** |  |  |  |  |  |  |
| Single | Reference |  |  |  |  |  |
| Married | -0.12 | 0.19 | 0.534 | 0.89 | 0.61 | 1.29 |
| Widowed or divorced | 0.24 | 0.35 | 0.490 | 1.27 | 0.64 | 2.54 |
| **Educational level** |  |  |  |  |  |  |
| High school or below | Reference |  |  |  |  |  |
| Bachelor | -0.45 | 0.22 | 0.043 | 0.64 | 0.41 | 0.99 |
| Postgraduate or above | -0.77 | 0.26 | 0.003 | 0.46 | 0.28 | 0.77 |
| **Occupation** |  |  |  |  |  |  |
| Government officials | Reference |  |  |  |  |  |
| Other governmental employees | -0.35 | 0.29 | 0.216 | 0.70 | 0.40 | 1.23 |
| Teachers or researchers | -0.18 | 0.25 | 0.486 | 0.84 | 0.51 | 1.38 |
| Police | -0.19 | 0.30 | 0.517 | 0.82 | 0.46 | 1.48 |
| Medical staff | -0.25 | 0.24 | 0.292 | 0.78 | 0.49 | 1.24 |
| **Position title** |  |  |  |  |  |  |
| Junior professional staff | Reference |  |  |  |  |  |
| Intermediate professional staff | -0.02 | 0.14 | 0.916 | 0.99 | 0.75 | 1.30 |
| Senior professional staff | -0.23 | 0.20 | 0.243 | 0.79 | 0.54 | 1.17 |
| **Physical activity** |  |  |  |  |  |  |
| <1/week | Reference |  |  |  |  |  |
| 1-2/week | -0.33 | 0.14 | 0.017 | 0.72 | 0.55 | 0.94 |
| >3/week | -0.49 | 0.14 | <0.001 | 0.62 | 0.47 | 0.80 |
| **Smoking** |  |  |  |  |  |  |
| Nonsmokers | Reference |  |  |  |  |  |
| Current Smokers | -0.30 | 0.18 | 0.090 | 0.74 | 0.52 | 1.05 |
| Former smokers | -0.17 | 0.39 | 0.662 | 0.84 | 0.39 | 1.81 |
| **Alcohol consumption** |  |  |  |  |  |  |
| Non-drinkers | Reference |  |  |  |  |  |
| Current drinkers | 0.36 | 0.14 | 0.009 | 1.44 | 1.09 | 1.90 |
| Former drinkers | -1.56 | 1.01 | 0.122 | 0.21 | 0.03 | 1.52 |
| **Family history of hypertension** |  |  |  |  |  |  |
| No | Reference |  |  |  |  |  |
| Yes | 0.24 | 0.11 | 0.035 | 1.27 | 1.02 | 1.58 |

*Note*: SBP, systolic blood pressure; DBP, diastolic blood pressure; BMI, body mass index; IRR, incidence rate ratios; CI, confidence interval; SE=standard error.

# 17. Supplemental Table

## **Table S17. Incidence rate ratios of hypertension by quartiles of positive and negative family-related life events**

| **Variable** | **Estimate** | **SE** | **P** | **IRR** | **95% CI** | |
| --- | --- | --- | --- | --- | --- | --- |
|  |  |  |  |  | **Lower** | **Upper** |
| Intercept | -15.82 | 0.94 | <0.001 | 0.00 | 0.00 | 0.00 |
| Age | 0.03 | 0.01 | 0.001 | 1.03 | 1.01 | 1.05 |
| Sex |  |  |  |  |  |  |
| Male | Reference |  |  |  |  |  |
| Female | -0.03 | 0.14 | 0.827 | 0.97 | 0.73 | 1.29 |
| BMI | 0.01 | 0.01 | 0.419 | 1.01 | 0.99 | 1.02 |
| SBP | 0.08 | 0.01 | <0.001 | 1.08 | 1.07 | 1.10 |
| DBP | 0.04 | 0.01 | <0.001 | 1.04 | 1.02 | 1.06 |
| **Total score of negative family-related life events** |  |  |  |  |  |  |
| Quartile 1 and 2 | Reference |  |  |  |  |  |
| Quartile 3 | 0.36 | 0.13 | 0.006 | 1.43 | 1.11 | 1.84 |
| Quartile 4 | 0.47 | 0.14 | 0.001 | 1.61 | 1.22 | 2.12 |
| **Total score of positive family-related life events** |  |  |  |  |  |  |
| Quartile 1 and 2 | Reference |  |  |  |  |  |
| Quartile 3 | -0.37 | 0.24 | 0.123 | 0.69 | 0.43 | 1.11 |
| Quartile 4 | -0.59 | 0.18 | 0.001 | 0.55 | 0.39 | 0.80 |
| **Marital status** |  |  |  |  |  |  |
| Single | Reference |  |  |  |  |  |
| Married | 0.00 | 0.19 | 0.984 | 1.00 | 0.69 | 1.47 |
| Widowed or divorced | 0.25 | 0.35 | 0.481 | 1.28 | 0.64 | 2.55 |
| **Educational level** |  |  |  |  |  |  |
| High school or below | Reference |  |  |  |  |  |
| Bachelor | -0.48 | 0.22 | 0.032 | 0.62 | 0.40 | 0.96 |
| Postgraduate or above | -0.76 | 0.26 | 0.003 | 0.47 | 0.28 | 0.77 |
| **Occupation** |  |  |  |  |  |  |
| Government officials | Reference |  |  |  |  |  |
| Other governmental employees | -0.33 | 0.29 | 0.251 | 0.72 | 0.41 | 1.26 |
| Teachers or researchers | -0.16 | 0.25 | 0.540 | 0.86 | 0.52 | 1.41 |
| Police | -0.18 | 0.30 | 0.546 | 0.83 | 0.46 | 1.50 |
| Medical staff | -0.20 | 0.24 | 0.402 | 0.82 | 0.51 | 1.31 |
| **Position title** |  |  |  |  |  |  |
| Junior professional staff | Reference |  |  |  |  |  |
| Intermediate professional staff | -0.04 | 0.14 | 0.802 | 0.96 | 0.73 | 1.27 |
| Senior professional staff | -0.27 | 0.20 | 0.170 | 0.76 | 0.52 | 1.12 |
| **Physical activity** |  |  |  |  |  |  |
| <1/week | Reference |  |  |  |  |  |
| 1-2/week | -0.33 | 0.14 | 0.018 | 0.72 | 0.55 | 0.95 |
| >3/week | -0.46 | 0.14 | 0.001 | 0.63 | 0.48 | 0.82 |
| **Smoking** |  |  |  |  |  |  |
| Nonsmokers | Reference |  |  |  |  |  |
| Current Smokers | -0.29 | 0.18 | 0.096 | 0.75 | 0.53 | 1.05 |
| Former smokers | -0.18 | 0.39 | 0.648 | 0.84 | 0.39 | 1.80 |
| **Alcohol consumption** |  |  |  |  |  |  |
| Non-drinkers | Reference |  |  |  |  |  |
| Current drinkers | 0.35 | 0.14 | 0.013 | 1.42 | 1.08 | 1.86 |
| Former drinkers | -1.50 | 1.01 | 0.138 | 0.22 | 0.03 | 1.61 |
| **Family history of hypertension** |  |  |  |  |  |  |
| No | Reference |  |  |  |  |  |
| Yes | 0.22 | 0.11 | 0.050 | 1.25 | 1.00 | 1.56 |

*Note*: SBP, systolic blood pressure; DBP, diastolic blood pressure; BMI, body mass index; IRR, incidence rate ratios; CI, confidence interval; SE=standard error.

# 18. Supplemental Table

## **Table S18. Incidence rate ratios of hypertension by quartiles of other personal life events**

| **Variable** | **Estimate** | **SE** | ***P*** | **IRR** | **95% CI** | |
| --- | --- | --- | --- | --- | --- | --- |
|  |  |  |  |  | **Lower** | **Upper** |
| Intercept | -16.22 | 0.95 | <0.001 | 0.00 | 0.00 | 0.00 |
| Age | 0.03 | 0.01 | <0.001 | 1.04 | 1.02 | 1.05 |
| **Sex** |  |  |  |  |  |  |
| Male | Reference |  |  |  |  |  |
| Female | 0.00 | 0.14 | 0.994 | 1.00 | 0.75 | 1.33 |
| BMI | 0.01 | 0.01 | 0.420 | 1.01 | 0.99 | 1.02 |
| SBP | 0.08 | 0.01 | <0.001 | 1.08 | 1.06 | 1.10 |
| DBP | 0.04 | 0.01 | <0.001 | 1.04 | 1.02 | 1.06 |
| **Total score of other personal life events** |  |  |  |  |  |  |
| Quartile 1 and 2 | Reference |  |  |  |  |  |
| Quartile 2 | 0.27 | 0.13 | 0.043 | 1.31 | 1.01 | 1.70 |
| Quartile 3 | 0.42 | 0.15 | 0.004 | 1.53 | 1.15 | 2.03 |
| **Marital status** |  |  |  |  |  |  |
| Single | Reference |  |  |  |  |  |
| Married | -0.03 | 0.18 | 0.879 | 0.97 | 0.68 | 1.39 |
| Widowed or divorced | 0.26 | 0.35 | 0.460 | 1.29 | 0.66 | 2.54 |
| **Educational level** |  |  |  |  |  |  |
| High school or below | Reference |  |  |  |  |  |
| Bachelor | -0.45 | 0.22 | 0.044 | 0.64 | 0.41 | 0.99 |
| Postgraduate or above | -0.77 | 0.26 | 0.003 | 0.46 | 0.28 | 0.76 |
| **Occupation** |  |  |  |  |  |  |
| Government officials | Reference |  |  |  |  |  |
| Other governmental employees | -0.33 | 0.29 | 0.250 | 0.72 | 0.41 | 1.26 |
| Teachers or researchers | -0.17 | 0.25 | 0.495 | 0.84 | 0.51 | 1.38 |
| Police | -0.18 | 0.30 | 0.541 | 0.83 | 0.46 | 1.50 |
| Medical staff | -0.24 | 0.24 | 0.323 | 0.79 | 0.49 | 1.26 |
| **Position title** |  |  |  |  |  |  |
| Junior professional staff | Reference |  |  |  |  |  |
| Intermediate professional staff | 0.01 | 0.14 | 0.949 | 1.01 | 0.76 | 1.33 |
| Senior professional staff | -0.22 | 0.20 | 0.277 | 0.81 | 0.55 | 1.19 |
| **Physical activity** |  |  |  |  |  |  |
| <1/week | Reference |  |  |  |  |  |
| 1-2/week | -0.32 | 0.14 | 0.022 | 0.73 | 0.55 | 0.96 |
| >3/week | -0.47 | 0.14 | 0.001 | 0.63 | 0.48 | 0.82 |
| **Smoking** |  |  |  |  |  |  |
| Nonsmokers | Reference |  |  |  |  |  |
| Current Smokers | -0.31 | 0.18 | 0.084 | 0.74 | 0.52 | 1.04 |
| Former smokers | -0.22 | 0.39 | 0.582 | 0.81 | 0.37 | 1.73 |
| **Alcohol consumption** |  |  |  |  |  |  |
| Non-drinkers | Reference |  |  |  |  |  |
| Current drinkers | 0.34 | 0.14 | 0.014 | 1.41 | 1.07 | 1.86 |
| Former drinkers | -1.52 | 1.01 | 0.131 | 0.22 | 0.03 | 1.57 |
| **Family history of hypertension** |  |  |  |  |  |  |
| No | Reference |  |  |  |  |  |
| Yes | 0.23 | 0.11 | 0.040 | 1.26 | 1.01 | 1.57 |

*Note*: SBP, systolic blood pressure; DBP, diastolic blood pressure; BMI, body mass index; IRR, incidence rate ratios; CI, confidence interval; SE=standard error.

# 19. Supplemental Table

## **Table S19. Incidence rate ratios of hypertension by quartiles of other positive and negative personal life events**

| **Variable** | **Estimate** | **SE** | **P** | **IRR** | **95% CI** | |
| --- | --- | --- | --- | --- | --- | --- |
|  |  |  |  |  | **Lower** | **Upper** |
| Intercept | -16.24 | 0.95 | <0.001 | 0.00 | 0.00 | 0.00 |
| Age | 0.03 | 0.01 | <0.001 | 1.03 | 1.02 | 1.05 |
| **Sex** |  |  |  |  |  |  |
| Male | Reference |  |  |  |  |  |
| Female | -0.01 | 0.14 | 0.970 | 0.99 | 0.75 | 1.32 |
| BMI | 0.01 | 0.01 | 0.429 | 1.01 | 0.99 | 1.02 |
| SBP | 0.08 | 0.01 | <0.001 | 1.08 | 1.07 | 1.10 |
| DBP | 0.04 | 0.01 | <0.001 | 1.04 | 1.02 | 1.06 |
| **Total score of other negative personal life events** | |  |  |  |  |  |
| Quartile 1 and 2 | Reference |  |  |  |  |  |
| Quartile 2 | 0.53 | 0.17 | 0.002 | 1.69 | 1.21 | 2.38 |
| Quartile 3 | 0.53 | 0.13 | <0.001 | 1.70 | 1.32 | 2.18 |
| **Total score of other positive personal life events** |  |  |  |  |  |  |
| Quartile 1, 2 and 3 | Reference |  |  |  |  |  |
| Quartile 4 | -0.21 | 0.15 | 0.173 | 0.81 | 0.60 | 1.10 |
| **Marital status** |  |  |  |  |  |  |
| Single | Reference |  |  |  |  |  |
| Married | -0.06 | 0.18 | 0.734 | 0.94 | 0.66 | 1.35 |
| Widowed or divorced | 0.20 | 0.35 | 0.556 | 1.23 | 0.62 | 2.41 |
| **Educational level** |  |  |  |  |  |  |
| High school or below | Reference |  |  |  |  |  |
| Bachelor | -0.44 | 0.22 | 0.050 | 0.65 | 0.42 | 1.00 |
| Postgraduate or above | -0.75 | 0.26 | 0.004 | 0.47 | 0.29 | 0.79 |
| **Occupation** |  |  |  |  |  |  |
| Government officials | Reference |  |  |  |  |  |
| Other governmental employees | -0.33 | 0.29 | 0.246 | 0.72 | 0.41 | 1.26 |
| Teachers or researchers | -0.17 | 0.25 | 0.506 | 0.84 | 0.51 | 1.39 |
| Police | -0.20 | 0.30 | 0.501 | 0.82 | 0.45 | 1.47 |
| Medical staff | -0.24 | 0.24 | 0.323 | 0.79 | 0.49 | 1.26 |
| **Position title** |  |  |  |  |  |  |
| Junior professional staff | Reference |  |  |  |  |  |
| Intermediate professional staff | -0.03 | 0.14 | 0.841 | 0.97 | 0.74 | 1.28 |
| Senior professional staff | -0.26 | 0.20 | 0.186 | 0.77 | 0.52 | 1.13 |
| **Physical activity** |  |  |  |  |  |  |
| <1/week | Reference |  |  |  |  |  |
| 1-2/week | -0.30 | 0.14 | 0.032 | 0.74 | 0.56 | 0.98 |
| >3/week | -0.45 | 0.14 | 0.001 | 0.64 | 0.49 | 0.83 |
| **Smoking** |  |  |  |  |  |  |
| Nonsmokers | Reference |  |  |  |  |  |
| Current Smokers | -0.30 | 0.18 | 0.087 | 0.74 | 0.52 | 1.04 |
| Former smokers | -0.24 | 0.39 | 0.541 | 0.79 | 0.37 | 1.69 |
| **Alcohol consumption** |  |  |  |  |  |  |
| Non-drinkers | Reference |  |  |  |  |  |
| Current drinkers | 0.34 | 0.14 | 0.014 | 1.41 | 1.07 | 1.86 |
| Former drinkers | -1.52 | 1.01 | 0.133 | 0.22 | 0.03 | 1.58 |
| **Family history of hypertension** |  |  |  |  |  |  |
| No | Reference |  |  |  |  |  |
| Yes | 0.23 | 0.11 | 0.042 | 1.26 | 1.01 | 1.57 |

*Note*: SBP, systolic blood pressure; DBP, diastolic blood pressure; BMI, body mass index; IRR, incidence rate ratios; CI, confidence interval; SE=standard error.

# 20. Supplemental Table

## **Table S20. Interactive and subgroup analysis hypertension IRRs and 95% CIs by total score of all life events**

| **Variable** | **Estimate** | **SE** | ***P*** | **IRR** | **95% CI** | | ***P* _interaction_** |
| --- | --- | --- | --- | --- | --- | --- | --- |
|  |  |  |  |  | **Lower** | **Upper** |  |
| **Age** |  |  |  |  |  |  | 0.711 |
| ≤30 | 0.01 | 0.02 | 0.587 | 1.01 | 0.97 | 1.05 |  |
| ≤40 | 0.01 | 0.01 | 0.315 | 1.01 | 0.99 | 1.04 |  |
| ≤60 | 0.03 | 0.02 | 0.088 | 1.03 | 1.00 | 1.06 |  |
| ≤50 | 0.02 | 0.02 | 0.322 | 1.02 | 0.98 | 1.07 |  |
| **Sex** |  |  |  |  |  |  | 0.636 |
| Male | 0.02 | 0.01 | 0.029 | 1.02 | 1.00 | 1.05 |  |
| Female | 0.01 | 0.01 | 0.262 | 1.01 | 0.99 | 1.04 |  |
| **BMI** |  |  |  |  |  |  | 0.263 |
| ≤18 | -0.02 | 0.08 | 0.782 | 0.98 | 0.84 | 1.14 |  |
| ≤24 | 0.02 | 0.01 | 0.088 | 1.02 | 1.00 | 1.04 |  |
| ≤30 | 0.02 | 0.01 | 0.287 | 1.02 | 0.99 | 1.04 |  |
| >30 | 0.06 | 0.03 | 0.059 | 1.06 | 1.00 | 1.12 |  |
| **Marital status** |  |  |  |  |  |  | 0.874 |
| Single | 0.04 | 0.02 | 0.076 | 1.04 | 1.00 | 1.09 |  |
| Married | 0.02 | 0.01 | 0.058 | 1.02 | 1.00 | 1.04 |  |
| Divorced or widowed | 0.04 | 0.06 | 0.530 | 1.04 | 0.93 | 1.16 |  |
| **Education level** |  |  |  |  |  |  | 0.967 |
| ≤High school | 0.02 | 0.03 | 0.612 | 1.02 | 0.95 | 1.09 |  |
| Bachelor | 0.02 | 0.01 | 0.016^b^ | 1.02 | 1.00 | 1.04 |  |
| ≥Postgraduate | 0.01 | 0.02 | 0.401 | 1.01 | 0.98 | 1.05 |  |
| **Occupation** |  |  |  |  |  |  | 0.678 |
| Government officials | 0.04 | 0.04 | 0.327 | 1.04 | 0.96 | 1.13 |  |
| Other governmental employees | 0.04 | 0.04 | 0.244 | 1.04 | 0.97 | 1.12 |  |
| Teachers or researchers | 0.03 | 0.02 | 0.073 | 1.03 | 1.00 | 1.07 |  |
| Police | 0.02 | 0.03 | 0.584 | 1.02 | 0.96 | 1.08 |  |
| Medical staff | 0.01 | 0.01 | 0.239 | 1.01 | 0.99 | 1.04 |  |
| **P****osition title** |  |  |  |  |  |  | 0.557 |
| Junior professional staff | 0.02 | 0.01 | 0.218 | 1.02 | 0.99 | 1.04 |  |
| Intermediate professional staff | 0.02 | 0.01 | 0.205 | 1.02 | 0.99 | 1.04 |  |
| Senior professional staff | 0.04 | 0.02 | 0.047 | 1.04 | 1.00 | 1.08 |  |
| **P****hysical activity** |  |  |  |  |  |  | 0.817 |
| <1/week | 0.01 | 0.01 | 0.284 | 1.01 | 0.99 | 1.03 |  |
| 1-2/week | 0.03 | 0.02 | 0.097 | 1.03 | 0.99 | 1.07 |  |
| >3/week | 0.02 | 0.02 | 0.144 | 1.03 | 0.99 | 1.06 |  |
| **Smoking** |  |  |  |  |  |  | 0.396 |
| Nonsmokers | 0.01 | 0.01 | 0.125 | 1.01 | 1.00 | 1.03 |  |
| Current Smokers | 0.04 | 0.02 | 0.033 | 1.04 | 1.00 | 1.08 |  |
| Former smokers | - | - | - | - | - | - |  |
| **A****lcohol consumption** |  |  |  |  |  |  | 0.469 |
| Non-drinkers | 0.01 | 0.01 | 0.170 | 1.01 | 0.99 | 1.03 |  |
| Current drinkers | 0.04 | 0.02 | 0.017^b^ | 1.04 | 1.01 | 1.07 |  |
| Former drinkers | - | - | - | - | - | - |  |
| **Family history of hypertension** | | |  |  |  |  | 0.421 |
| With family history of hypertension | 0.02 | 0.01 | 0.055 | 1.02 | 1.00 | 1.04 |  |
| Without family history of hypertension | 0.02 | 0.01 | 0.204 | 1.02 | 0.99 | 1.04 |  |

Note: ^a^The Bonferroni-corrected significance levels for sex and family history of hypertension were 0.05/2=0.025. ^b^The Bonferroni-corrected significance levels for marital status, education level, occupation, position title, physical activity, smoking, and alcohol consumption were 0.05/3=0.017. ^c^The Bonferroni-corrected significance levels for age and BMI were 0.05/4=0.0125. ^d^The Bonferroni-corrected significance levels for occupation were 0.05/5=0.01. SE=standard error; IRR=incidence rate ratio; CI= confidence interval; BMI= body mass index. SBP, systolic blood pressure; DBP, diastolic blood pressure; BMI, body mass index; IRR, incidence rate ratios; CI, confidence interval; SE=standard error.

# 21. Supplemental Table

## **Table S21. Interactive and subgroup analysis hypertension IRRs and 95% CIs by positive and negative life events**

| **Subgroups** | **IRR** | **95% CI** | | ***P*** | ***P* _interaction_** | **IRR** | **95% CI** | | ***P*** | ***P* _interaction_** |
| --- | --- | --- | --- | --- | --- | --- | --- | --- | --- | --- |
|  |  | **Upper** | **Lower** |  |  |  | **Upper** | **Lower** |  |  |
| **Age** |  |  |  |  | 0.713 |  |  |  |  | 0.390 |
| ≤30 | 0.85 | 0.75 | 0.97 | 0.013 |  | 1.06 | 1.02 | 1.11 | 0.006^c^ |  |
| ≤40 | 0.87 | 0.79 | 0.96 | 0.006^c^ |  | 1.05 | 1.02 | 1.08 | 0.001^c^ |  |
| ≤60 | 0.94 | 0.79 | 1.11 | 0.445 |  | 1.04 | 1.00 | 1.08 | 0.028 |  |
| ≤50 | 0.98 | 0.82 | 1.17 | 0.841 |  | 1.03 | 0.98 | 1.09 | 0.257 |  |
| **Sex** |  |  |  |  | 0.075 |  |  |  |  | 0.974 |
| Male | 0.98 | 0.90 | 1.06 | 0.612 |  | 1.03 | 1.01 | 1.06 | 0.013^a^ |  |
| Female | 0.82 | 0.74 | 0.91 | <0.001^a^ |  | 1.06 | 1.03 | 1.08 | <0.001^a^ |  |
| **BMI** |  |  |  |  | 0.236 |  |  |  |  | 0.449 |
| ≤18 | 1.00 | 0.70 | 1.43 | 0.996 |  | 0.97 | 0.77 | 1.21 | 0.772 |  |
| ≤24 | 0.84 | 0.76 | 0.94 | 0.001^c^ |  | 1.05 | 1.03 | 1.08 | <0.001^c^ |  |
| ≤30 | 0.93 | 0.84 | 1.02 | 0.125 |  | 1.04 | 1.00 | 1.07 | 0.029 |  |
| >30 | 1.02 | 0.85 | 1.23 | 0.816 |  | 1.07 | 0.98 | 1.16 | 0.111 |  |
| **Marital status** |  |  |  |  | 0.673 |  |  |  |  | 0.453 |
| Single | 0.95 | 0.79 | 1.15 | 0.626 |  | 1.06 | 1.00 | 1.12 | 0.037 |  |
| Married | 0.89 | 0.83 | 0.96 | 0.001^b^ |  | 1.05 | 1.03 | 1.07 | <0.001^b^ |  |
| Divorced or widowed | 1.06 | 0.56 | 2.00 | 0.866 |  | 1.04 | 0.93 | 1.16 | 0.543 |  |
| **Education level** |  |  |  |  | 0.670 |  |  |  |  | 0.656 |
| ≤High school | 1.01 | 0.73 | 1.40 | 0.935 |  | 1.02 | 0.93 | 1.11 | 0.704 |  |
| Bachelor | 0.90 | 0.83 | 0.98 | 0.014^b^ |  | 1.05 | 1.02 | 1.07 | <0.001^b^ |  |
| ≥Postgraduate | 0.89 | 0.80 | 1.00 | 0.045 |  | 1.05 | 1.01 | 1.09 | 0.012^b^ |  |
| **Occupation** |  |  |  |  | 0.606 |  |  |  |  | 0.365 |
| Government officials | 1.08 | 0.82 | 1.43 | 0.574 |  | 1.03 | 0.93 | 1.15 | 0.544 |  |
| Other governmental employees | 1.08 | 0.85 | 1.36 | 0.534 |  | 1.03 | 0.94 | 1.13 | 0.478 |  |
| Teachers or researchers | 0.96 | 0.84 | 1.09 | 0.490 |  | 1.05 | 1.01 | 1.09 | 0.021 |  |
| Police | 1.11 | 0.85 | 1.45 | 0.452 |  | 1.00 | 0.93 | 1.08 | 0.946 |  |
| Medical staff | 0.84 | 0.77 | 0.92 | <0.001^d^ |  | 1.05 | 1.03 | 1.08 | <0.001^d^ |  |
| **Position title** |  |  |  |  | 0.672 |  |  |  |  | 0.585 |
| Junior professional staff | 0.89 | 0.81 | 0.97 | 0.010^b^ |  | 1.04 | 1.02 | 1.07 | 0.002^b^ |  |
| Intermediate professional staff | 0.91 | 0.82 | 1.02 | 0.094 |  | 1.04 | 1.01 | 1.08 | 0.012^b^ |  |
| Senior professional staff | 0.89 | 0.73 | 1.08 | 0.244 |  | 1.07 | 1.02 | 1.11 | 0.004^b^ |  |
| **Physical activity** |  |  |  |  | 0.610 |  |  |  |  | 0.608 |
| <1/week | 0.89 | 0.82 | 0.97 | 0.006^b^ |  | 1.04 | 1.02 | 1.07 | 0.002^b^ |  |
| 1-2/week | 0.90 | 0.78 | 1.04 | 0.144 |  | 1.06 | 1.02 | 1.10 | 0.005^b^ |  |
| >3/week | 0.96 | 0.84 | 1.10 | 0.555 |  | 1.04 | 1.00 | 1.07 | 0.058^b^ |  |
| **Smoking** |  |  |  |  | 0.091 |  |  |  |  | 0.614 |
| Nonsmokers | 0.89 | 0.83 | 0.96 | 0.001^b^ |  | 1.05 | 1.02 | 1.07 | <0.001^b^ |  |
| Current Smokers | 1.01 | 0.86 | 1.20 | 0.861 |  | 1.05 | 1.00 | 1.10 | 0.045 |  |
| Former smokers | 0.00 | 0.00 | Inf | 1.000^b^ |  | 0.43 | 0.00 | Inf | 1.000^b^ |  |
| **Alcohol consumption** |  |  |  |  | 0.446 |  |  |  |  | 0.642 |
| Non-drinkers | 0.89 | 0.83 | 0.96 | 0.003^b^ |  | 1.04 | 1.02 | 1.06 | <0.001^b^ |  |
| Current drinkers | 0.92 | 0.82 | 1.04 | 0.185 |  | 1.06 | 1.03 | 1.10 | 0.001^b^ |  |
| Former drinkers | - | - | - | - |  | - | - | - | - |  |
| **Family history of hypertension** | |  |  |  | 0.293 |  |  |  |  | 0.865 |
| With family history of hypertension | 0.88 | 0.81 | 0.96 | 0.003^a^ |  | 1.05 | 1.03 | 1.08 | <0.001^a^ |  |
| Without family history of hypertension | 0.93 | 0.84 | 1.02 | 0.136 |  | 1.04 | 1.01 | 1.07 | 0.020^a^ |  |

Note: ^a^The Bonferroni-corrected significance levels for sex and family history of hypertension were 0.05/2=0.025. ^b^The Bonferroni-corrected significance levels for marital status, education level, occupation, position title, physical activity, smoking, and alcohol consumption were 0.05/3=0.017. ^c^The Bonferroni-corrected significance levels for age and BMI were 0.05/4=0.0125. ^d^The Bonferroni-corrected significance levels for occupation were 0.05/5=0.01. SE=standard error; IRR=incidence rate ratio; CI= confidence interval; BMI= body mass index. SBP, systolic blood pressure; DBP, diastolic blood pressure; BMI, body mass index; IRR, incidence rate ratios; CI, confidence interval; SE=standard error.

# 22. Supplemental Table

## **Table S22. Sensitivity analysis**

| **Variable** | **Estimate** | **SE** | **P** | **IRR** | **95% CI** | |
| --- | --- | --- | --- | --- | --- | --- |
|  |  |  |  |  | **Lower** | **Upper** |
| **The full life events scale** |  |  |  |  |  |  |
| **All life events** |  |  |  |  |  |  |
| Total score of all life events | 0.02 | 0.01 | 0.015 | 1.02 | 1.00 | 1.04 |
| **Positive and negative life events*** |  |  |  |  |  |  |
| Score of all positive life events | -0.11 | 0.03 | 0.002 | 0.90 | 0.84 | 0.96 |
| Score of all negative life events | 0.04 | 0.01 | <0.001 | 1.05 | 1.03 | 1.06 |
| **Work-related life events**** |  |  |  |  |  |  |
| **All work-related life events** |  |  |  |  |  |  |
| Total score of work-related life events | 0.08 | 0.02 | 0.001 | 1.08 | 1.03 | 1.13 |
| **Positive and negative work-related life events*** |  |  |  |  |  |  |
| Score of positive work-related life events | -0.14 | 0.08 | 0.076 | 0.87 | 0.74 | 1.02 |
| Score of negative work-related life events | 0.12 | 0.02 | <0.001 | 1.12 | 1.07 | 1.18 |
| **Family-related life events**** |  |  |  |  |  |  |
| **All family-related life events** |  |  |  |  |  |  |
| Total score of family-related life events | 0.00 | 0.02 | 0.768 | 1.00 | 0.97 | 1.04 |
| **Positive and Negative family-related life events*** |  |  |  |  |  |  |
| Score of positive family-related life events | -0.13 | 0.05 | 0.004 | 0.87 | 0.80 | 0.96 |
| Score of negative family-related life events | 0.04 | 0.02 | 0.026 | 1.04 | 1.00 | 1.08 |
| **Other personal life events**** |  |  |  |  |  |  |
| **All other personal life events** |  |  |  |  |  |  |
| Total score of other personal life events | 0.07 | 0.02 | 0.001 | 1.08 | 1.03 | 1.13 |
| **Other positive and negative personal life events*** |  |  |  |  |  |  |
| Score of other positive personal life events | -0.02 | 0.08 | 0.799 | 0.98 | 0.85 | 1.14 |
| Score of other negative personal life events | 0.09 | 0.03 | <0.001 | 1.10 | 1.04 | 1.15 |

***Note:*** ^*^The score of positive and negative life events were added concurrently into the Poisson models. **^**^**Hypertension incidence rate ratios of work-related, family-related, and other personal life events were calculated in different Poisson models. IRR, incidence rate ratios; CI, confidence interval; SE=standard error.
